# Coagulation potential and the integrated omics of extracellular vesicles from COVID-19 positive patient plasma

**DOI:** 10.1038/s41598-022-26473-8

**Published:** 2022-12-23

**Authors:** Saini Setua, Kiruphagaran Thangaraju, Monika Dzieciatkowska, Rebecca B. Wilkerson, Travis Nemkov, Derek R. Lamb, Yutaka Tagaya, Tori Boyer, Tobi Rowden, Allan Doctor, Angelo D’Alessandro, Paul W. Buehler

**Affiliations:** 1 Department of Pediatrics, Center for Blood Oxygen Transport and Hemostasis, University of Maryland School of Medicine, Baltimore, MD USA; 2 Department of Biochemistry and Molecular Genetics, University of Colorado, Denver-Anschutz Medical Campus, 12801 East 17th Ave., Aurora, CO 80045 USA; 3 Division of Virology, Pathogenesis and Cancer, Institute of Human Virology, University of Maryland School of Medicine, Baltimore, MD USA; 4 Department of Pathology, University of Maryland School of Medicine, Baltimore, MD 21201 USA

**Keywords:** Haematological diseases, Biomarkers, Thrombosis, Metabolomics, Proteomics

## Abstract

Extracellular vesicles (EVs) participate in cell-to-cell communication and contribute toward homeostasis under physiological conditions. But EVs can also contribute toward a wide array of pathophysiology like cancer, sepsis, sickle cell disease, and thrombotic disorders. COVID-19 infected patients are at an increased risk of aberrant coagulation, consistent with elevated circulating levels of ultra-high molecular weight VWF multimers, D-dimer and procoagulant EVs. The role of EVs in COVID-19 related hemostasis may depend on cells of origin, vesicular cargo and size, however this is not well defined. We hypothesized that the procoagulant potential of EV isolates from COVID-19 (+) patient plasmas could be defined by thrombin generation assays. Here we isolated small EVs (SEVs) and large EVs (LEVs) from hospitalized COVID-19 (+) patient (n = 21) and healthy donor (n = 20) plasmas. EVs were characterized by flow cytometry, Transmission electron microscopy, nanoparticle tracking analysis, plasma thrombin generation and a multi-omics approach to define coagulation potential. These data were consistent with differences in EV metabolite, lipid, and protein content when compared to healthy donor plasma isolated SEVs and LEVs. Taken together, the effect of EVs on plasma procoagulant potential as defined by thrombin generation and supported by multi-omics is enhanced in COVID-19. Further, we observe that this effect is driven both by EV size and phosphatidyl serine.

## Introduction

Coronavirus disease (COVID-19), caused by severe acute respiratory syndrome coronavirus 2 (SARS-CoV-2), has affected the lives of millions of individuals worldwide^1,2^. COVID-19 pneumonia and acute respiratory distress syndrome are associated with features of immunothrombosis mediated by proinflammatory cytokines, neutrophils, platelets, endothelial cell activation, and microvascular thrombosis^3^. Studies suggest that coagulopathy and endothelial dysfunction are important pathophysiological events in COVID-19 infection^3,4^. Elevated levels of Von Willebrand factor (VWF), fibrinogen, and the fibrin degradation product D-dimer, along with a mildly prolonged prothrombin time (PT) and thromboelastographic data that indicates clot stability, all support a COVID-19 specific coagulopathy hypothesis^5–8^. Increased thrombin generation (TG) and a reduced availability of plasmin may also play a significant role in COVID-19 associated thrombosis and thrombolysis^9–11^. Circulating markers are important for the identification, diagnosis, and treatment of aberrant coagulation in COVID-19^12^. Further, vesicles shed from cellular membranes can function as indicators or modifiers of disease and their accumulation within the plasma of COVID-19 (+) patients are potential sources for enhancing coagulation risk.

Extracellular vesicles (EVs) are membrane surrounded structures released by most cell types and characterized by small dimensions and heterogeneity^13–15^. They are classified in to three main groups by size (diameter): exosomes (30–150 nm), microvesicles (100–1000 nm), and apoptotic bodies (1000–3000 nm)^16,17^. Small EVs (SEVs, demonstrate mean sizes within the exosome size range) are the smallest vesicles released by the fusion of multivesicular bodies containing intraluminal vesicles with the plasma membrane. Large EVs (LEVs, also referred to as microvesicles) are vesicular structures shed by outward blebbing of the plasma membrane. EVs are retrieved in blood and other body fluids including saliva, urine, semen, breast milk, sputum, cerebrospinal fluid, and nasal fluid^13,14^. Extracellular vesicles are found in circulation and contain cell-derived biomolecules including proteins, lipids, DNA, RNA, and metabolites that can play a role in the pathogenesis of various diseases, including viral infections^18,19^. Studies have shown that tissue factor (TF) and phosphatidylserine (PS) are carried mostly by LEVs; therefore, they are the most procoagulant of the EVs. LEVs facilitate assembly of tenase and prothrombinase complexes on PS, thus mediating procoagulant activities and promoting TG.

A broad range of EV functions are described during the progression of COVID-19 infection. It is suggested that EVs might mediate viral cell entry by regulating angiotensin converting enzyme 2, a key protein recognized by SARS-CoV2^20–23^. Additionally, in vitro studies suggest that EVs can carry SARS-CoV2 spike proteins and act as a decoy for neutralizing antibodies^24^. Several omics-driven approaches to the evaluation of EVs from COVID-19 (+) patient plasma have predominantly identified coagulation and inflammation specific markers^25–29^. Flow cytometric assays identify increased platelet- and endothelium-derived EVs from COVID-19 (+) patient plasma based on CD41 and CD31 surface marker identification, respectively^30^. The presence of TF on the surface of EVs from COVID-19 patient plasma is reported to correlate with increased serum levels of inflammatory cytokines^31^. Further, an increase in EVs with both TF content and activity in COVID-19 (+) patients is reported, suggesting an ability to bind FVIIa and initiate coagulation^32–34^. Fractionation and characterization of EVs suggests a size dependent difference in molecular cargo that contribute to coagulopathy. The SEVs and LEVs from COVID-19 (+) patient plasma indicate that the mean size and concentration of both EV populations increase in circulation with disease severity and that prothrombotic factor content in larger EVs is greater^34^. To date, only PS-exposing EVs derived from platelets are reported in COVID-19 (+) patients^35^. Additionally, the loss of endothelial thrombomodulin (TM) and an increase in its circulating levels are reported to associate with reduced survival of COVID-19 (+) patients. However, the presence and function of TM in COVID-19 EVs is not known.

Given the procoagulant nature of EVs generated during disease and their role in thrombosis^36,37^, we hypothesized that SEVs and LEVs from COVID-19 (+) patients and healthy donors would exert differential effects on thrombin-initiated coagulation. Further, we suggest that the metabolome, lipidome, and proteome of circulating EVs isolated from the plasma of hospitalized COVID-19 (+) patients describes the signature that underpins their coagulopathic potential. To this end, we isolated SEVs and LEVs from COVID-19 (+) patient plasmas and compared them to EV isolates from healthy donors. Size distribution and EV markers were characterized by flow cytometry, nanoparticle tracking analysis (NTA), and transmission electron microscopy (TEM). Further, we performed a comprehensive analysis of the metabolic, lipid, protein compositions in both SEVs and LEVs and studied their effect on TG.

## Materials and methods

### Consent

This study was approved by the University of Maryland Institutional Review Board (protocol number: HP-00091732) and conducted in accordance with the 2003 Helsinki Declaration. Written informed consent was obtained from all enrolled patients.

### Patients

Blood samples were obtained from consenting COVID-19 (+) patients (n = 21) at the time of hospitalization. Diagnosis for COVID-19 was based on a positive SARS-CoV-2 RT-PCR test. Platelet poor plasma from age- and sex-matched healthy donors (n = 20) was obtained from Innovative Research, Inc. (Novi, MI, USA). All patient data was obtained from chart-based review at the time of hospital admission and throughout their hospital stay. Healthy donor plasma was obtained to approximate the demographics of patients included in this study.

### Sample collection and handling for research-based assays

All blood samples (4 mL) were collected from COVID-19 (+) patients into sodium citrate (0.109 M, 3.2%) Vacutainer blood collection tubes (Becton Dickenson, Franklin Lakes, NJ, USA) within the initial 24 h of admission and processed to platelet poor plasma. Samples were centrifuged at 800×*g* for 10 min to separate the RBCs and plasma was again centrifuged at 2500×*g* for 10 min and resulting plasma was analyzed to confirm removal of platelets using a hematology analyzer (Supplementary Fig. S1). Plasma was then aliquoted as 300–500 µL volumes into multiple Eppendorf tubes and frozen for isolation and analysis of EVs. Healthy donor blood samples were collected in sodium citrate tubes, processed to platelet poor plasma as mentioned above and frozen as 500 µL aliquots. Each aliquot of frozen plasma was only used one time and not refrozen for use in any other assays.

### Extracellular vesicle depleted pooled human plasma preparation

Pooled human platelet poor plasma (Apheresis derived sodium citrate) was purchased from Innovative Research, Inc. (Novi, MI, USA) and used for EV depleted plasma preparation. Plasma was centrifuged at 3200×*g* for 10 min to remove any debris and partly purified plasma was ultracentrifuged at 100,000×*g* for 2 h. Clear plasma was collected and further sterile filtered using 0.1 µm syringe filter (Minisart, Sartorius Stedim Biotech, Göttingen, Germany) and stored at −80 °C^38^.

### Small extracellular vesicle isolation

EVs that ranged in size from 100 to 150 nm are referred to herein as small extracellular vesicles (SEVs) and were isolated from COVID-19 (+) patients and healthy donor plasmas (500 µL) using a Total Exosome Isolation Kit (Invitrogen Cat# 4484450, California, USA), according to the manufacturer’s protocol. Briefly, platelet poor plasma was centrifuged at 2500×*g* for 20 min and further at 10,000×*g* for 20 min. The exosome isolation reagent and PBS was added to plasma samples and incubated on ice for 30 min and the mixture was centrifuged for 5 min at 10,000×*g*. The pellet was washed with 0.1 µm filtered PBS for 5 min at 10,000×*g* and finally dissolved in an appropriate volume of 0.1 µm filtered PBS.

## Large extracellular vesicle isolation

EVs that ranged in size from 200 to 600 nm are referred to herein as large extracellular vesicles (LEVs) and were isolated from an additional aliquot of the same healthy donor or COVID-19 (+) patient plasma. Samples were centrifuged for 15 min at 1500×*g*, followed by 3200×*g* for 15 min at room temperature to sediment out larger cell debris and the cell free plasma supernatant was collected for LEV isolation. Collected plasma was then centrifuged for 1 h at 20,000×*g*, 4 °C and the supernatant was discarded. The LEV pellet was washed with 1 mL of 0.1 µm filtered PBS and resuspended in the same buffer for further analyses.

### Quantification of isolated extracellular vesicles

Intact SEVs and LEVs were resuspended in PBS and used in our thrombin generation assay. Protein quantification of the SEVs and LEVs was performed using the Micro BCA Protein Assay Reagent Kit based on the manufacturer’s instructions (Thermo Scientific, Waltham, MA, USA). Briefly, assay reagents were mixed and incubated at room temperature for 1 min followed by addition of samples and incubation for 30 min at room temperature and read at 562 nm using synergy HTX reader (BioTek instruments, Winooski, Vermont, USA). To check the consistency, protein quantification of isolated SEVs and LEVs was also done with NanoDrop One spectrophotometer (Thermo Scientific, Waltham, MA, USA). Sample absorbance at 280 nm was used for protein quantification, using PBS as a blank. Both the techniques provided similar values, so NanoDrop was used for further measurements.

### Residual platelet count measurements

Residual platelets in platelet poor plasma from healthy donors, COVID-19 (+) patient and pooled plasma for EV depleted plasma, SEV and LEV preparations were quantified using ABX Pentra 60 C^+^ Hematology analyzer (HORIBA Instruments Inc., Kyoto, Japan). Briefly, 60 µL of plasma samples after different centrifugation steps were used for the analyses. 200 µg/mL of SEVs and LEVs were added to EV depleted plasma and used for the measurements. Further, residual platelets were evaluated by flow cytometry. Briefly, healthy plasma, 800×*g* supernatant, 3200×*g* supernatant and 10,000×*g* supernatants were analyzed by flow cytometry using CD41 as marker for platelets (Fig. S1B).

### Dynamic light scattering

The hydrodynamic size of isolated SEVs and LEVs were determined by the dynamic light scattering (DLS) principle using a Zetasizer (Nano ZS, Malvern Instruments, Malvern, UK).

### Transmission electron microscopy

Isolated SEVs and LEVs from COVID-19 (+) patient and healthy donor samples were evaluated by transmission electron microscopy (TEM) to visualize their size and morphology. Briefly, 10 μL of SEV and LEV suspensions were added to Formvar/carbon coated 200 mesh copper grids and allowed to dry for 2 min at room temperature and the excess suspension was wiped off with Whatman filter paper. Samples were then washed briefly with nanopure water and stained with 2% aqueous uranyl acetate (10 μL) for 2 min. The stained SEVs and LEVs were observed using a transmission electron microscope (Hitachi HT7800, Tokyo (HQ) Japan) at an acceleration voltage of 60 kV.

### Antibodies

The following list of antibodies were used in the flowcytometry analysis and TG assays: Alexa Fluor 647 conjugated anti-CD63 (Clone: H5C6, Cat # 353015), BV421 conjugated anti-CD41 (Clone: HIP8, Cat # 303729), PE dazzle 594 conjugated anti-CD235a (Clone: HI264, Cat # 349119), BV605 conjugated anti-CD31 (Clone: WM59, Cat # 303121), and PE conjugated anti-CD62P (Clone: AK4, Cat # 304905), Percp/cyanine5.5 Annexin V (Cat # 640935), Purified Anti-human CD142 (Clone: NY2, Cat # 365202), Purified anti-human CD141 (Thrombomodulin) Antibody (Clone: M80, Cat # 344102), Purified anti-human CD41 Antibody (Clone: HIP8, Cat # 303702), Purified anti-human CD235a (Glycophorin A) Antibody (Clone: HI264, Cat # 349102), Purified anti-human CD31 Antibody (Clone: WM59, Cat # 303101) all from Biolegend (San Diego, California, USA) and Alexa Fluor 488 conjugated anti-CD81 (Cat # FAB4615G), Mouse IgG_1_ Alexa Fluor® 647-conjugated Antibody (Cat # IC002R, Isotype Control), Mouse IgG2B Alexa Fluor® 488-conjugated Isotype Control (Cat # IC0041G) from R&D Systems, Minneapolis, USA.

### Flow cytometric characterization of SEVs and LEVs

Flow cytometry analysis was performed to classify the cellular origin of purified SEVs and LEVs as platelet, red blood cell, or endothelial cell derived, based on their surface markers. SEVs and LEVs were stained for 45 min at ambient temperature in the dark with a combination of Alexa Fluor 647 conjugated anti-CD63, Alexa Fluor 488 conjugated anti-CD81, BV421 conjugated anti-CD41, PE dazzle 594 conjugated anti-CD235a, BV605 conjugated anti-CD31, and PE conjugated anti-CD62P antibodies. Antibody solutions were centrifuged at 17,000×*g* for 10 min at 4 °C before incubation to avoid artefacts caused by antibody aggregates^38^. After incubation, SEVs and LEVs were resuspended in 500 μL particle free PBS and analyzed. Negative controls included buffer alone and unstained SEV and LEV samples. Single staining for specific antibodies was performed to determine the background. Each markers expression was represented as a histogram and normalized to mode. Forward scatter (FSC) and side scatter (SSC) were set to gate the SEV and LEV population. Calibration was done with flow cytometry sub-micron particle size reference kit (0.1, 0.2, 0.5 µm; cat # F13839, ThermoFisher scientific) according to manufacturer instruction. The SEV gate was set above the 200 nm particle distribution which includes the 100 nm and 200 nm beads clouds and gate for LEVs was set above the 500 nm bead population to include the distribution of all other beads, as shown in Fig. S2A. A detergent lysis control was performed to confirm the purity of the intact EVs signals. Both SEVs and LEVs were treated with 0.25% TritonX-100 during the staining process (CD63) (Fig. S3A). Control experiments for flowcytometric characterization was performed with buffer control, isotype control, and single staining of EVs characterization markers (CD63 and CD81) (Fig. S3B).

To evaluate the true expression of cell specific EVs, a blocking (cold inhibition) experiment was performed. Excess specific unlabeled antibodies (20X) were used to block the epitopes on the EV surface for 30 min incubation at room temperature and then incubated with fluorescently labeled antibody for 30 min at room temperature and analyzed as compared to the single stained samples (Fig. S4). All samples were evaluated using a BD FACSAria Flow cytometer (Becton Dickenson, Vancouver BC, Canada) and acquired data was analyzed using FlowJo 10.8.0 software (Becton Dickenson, Vancouver BC, Canada).

### Western blot analyses

The SEV, LEV and EDP proteins were extracted in RIPA buffer (Millipore Sigma, Burlington, MA, USA) and denatured in 4× Laemmli sample buffer (BIO-RAD, Hercules, CA, USA) at 99 °C for 10 min and 100 μg was resolved in 4% to 15% Tris glycine gel (Mini-PROTEAN^®^ TGX™ Precast Protein Gels, BIO-RAD, Hercules, CA, USA) for approximately 90 min at constant voltage (100 V). Proteins were transferred to PVDF membranes (Immobilon^®^-FL, Millipore Sigma, Burlington, MA, USA) using wet blot module (BIO-RAD, Hercules, CA, USA) for 90 min at fixed voltage (100 V), according to the manufacturer's instruction. Membranes were then washed with Tris-buffered saline with 0.1% Tween^®^ 20 Detergent (TBST) for 5 min and blocked for 1 h in 5% bovine serum albumin (BSA; Sigma-Aldrich, St Louis, MO, USA). Western blots were performed with rabbit anti-human CD63 (CST-52090S) and CD81 (CST-52892S) (Cell Signaling Technology, Danvers, MA, USA) at a dilution of 1:1000. The goat anti-rabbit IRDye^®^ 680RD secondary antibody (LI-COR, Lincoln, Nebraska, USA) was used at a dilution of 1:5000. Subsequent visualization was obtained using Odyssey CLx Imager (LI-COR, Lincoln, Nebraska, USA).

### Thrombin generation

TG was performed based on the assay originally developed by H. C. Hemker^39^ with modifications. Briefly, concentration (50, 100, 200 µg protein/mL) dependent SEV and LEV fractions were incubated with 70 µL of EV depleted human platelet poor pooled plasma (EDP). Samples were supplemented with buffer (150 mM NaCl, 20 mM HEPES at pH 7.5) and mixed with thrombin specific substrate, Z-Gly-Gly-Arg-AMC (Bachem, Bubendorf, Switzerland) at 3.08 mM final concentration. AMC fluorophore was added for the calibration measurement. The reaction was started by adding an activator solution comprised of a final concentration of 2 pM tissue factor (Diagnostica Stago, Parsippany, NJ, USA), 0.7 µg/mL of tissue plasminogen activator (tPA) (Sigma-Aldrich, St. Louis, MO, USA) and 16 mM CaCl_2_. Calculation and analysis of the TG curve was performed as previously described^40,41^. TM and annexin V blocking experiments were performed using SEV and LEV isolates (100 µg protein/mL) incubated with 10 µg/mL of anti-human CD141 (TM) antibody and 0.1, 0.5, 2.5 µg/mL annexin V (Biolegend, San Diego, California, USA) at ambient temperature for 1 h. The TG assay was carried out as described above.

### Tissue factor activity

Tissue factor (TF) activity was measured using a human tissue factor activity assay kit (ab108906, Abcam, Cambridge, United Kingdom) with slight modifications to the manufactures protocol. 200 µg protein/mL SEV or LEV was incubated with the assay mixture overnight at 4 °C followed by a 30 min incubation at 37 °C. After addition of FXa substrate readings were recorded at 405 nm every 2 min for 1 h at 37 °C using synergy HTX reader (BioTek instruments, Winooski, Vermont, USA). The assay modifications were specific to the duration of mixture incubation being extended to overnight for the purposes of this study.

### Metabolomics and lipidomics

High-throughput metabolomics analysis was performed on SEVs and LEVs isolated from healthy donor and COVID-19 (+) patient plasmas. Samples were thawed on ice and metabolites or lipids extracted by adding −20 °C methanol:acetonitrile:water (5:3:2 v/v/v) or methanol to each tube, respectively—at a 1:9 sample:extraction solution ratio (v/v), followed by resting for 20 min at −20 °C and centrifugation at 4 °C. All extracts were analyzed twice (20 µL injection each) by ultra-high-performance liquid chromatography using a UHPLC Vanquish coupled with a Q Exactive mass spectrometer (both from ThermoFisher Scientific Inc., San Jose, CA, USA) using both negative and positive polarity modes. For each method, the UHPLC utilized an Acquity HSS column at a flow rate increasing from 0.3 to 0.4 mL/min or 0.325 to 0.4 mL/min for 17 or 15 min for lipidomics, or at a flow rate of 450 µL/min on a Kinetex C18 column (150 × 2.1 mm, 1.7 μm, Phenomenex, Torrance, CA, USA) using 5-min gradients in positive and negative ion polarity modes for metabolomics, respectively, as described previously^42,43^.

### Proteomics

Protein pellets from metabolomics/lipidomics samples were digested in an S-Trap filter (Protifi, Huntington, NY), following the manufacturer’s procedure. Briefly, ~ 50 μg of protein were first mixed with 5% SDS. Samples were reduced with 10 mM dithiothreitol at 55 °C for 30 min, cooled to room temperature, and then alkylated with 25 mM iodoacetamide in the dark for 30 min. Phosphoric acid was then added to a final concentration of 1.2% followed by 6 volumes of binding buffer (90% methanol; 100 mM triethylammonium bicarbonate (TEAB); pH 7.1). After gentle mixing, the protein solution was loaded onto an S-Trap filter, centrifuged (2000×*g*; 1 min), and the flow-through collected and reloaded onto the filter. This step was repeated three times, and then the filter was washed with 200 μL of binding buffer 3 times. Finally, 1 μg of sequencing-grade trypsin and 150 μL of digestion buffer (50 mM TEAB) were added onto the filter and digested at 47 °C for 1 h. To elute peptides, three step-wise buffers were applied, with 200 μL of each with one more repeat; these included 50 mM TEAB, 0.2% formic acid in water, and 50% acetonitrile and 0.2% formic acid in water. The peptide solutions were pooled, lyophilized, and resuspended in 0.1% formic acid.

Samples (200 ng each) were loaded onto individual Evotips for desalting and then washed with 20 μL 0.1% formic acid followed by adding 100 μL of storage solvent (0.1% formic acid) to keep the Evotips wet until analysis. The Evosep One system was coupled to the timsTOF Pro mass spectrometer (Bruker Daltonics, Bremen, Germany). Data were collected over an m/z range of 100–1700 for MS and MS/MS on the timsTOF Pro instrument using an accumulation and ramp time of 100 ms. Post processing was performed with PEAKS studio (Version X+, Bioinformatics Solutions Inc., Waterloo, ON). Pathway analyses were performed with DAVID software and Ingenuity Pathway Analysis.

### Statistical analysis

All statistical analyses and graphing of data were performed using GraphPad Prism software (version 9.2.0). Comparisons across groups were analyzed with a one-way-ANOVA and Tukey’s multiple comparison test. Lipidomic data analysis was performed using Maven (1.4.20-dev-772), and quality controls were maintained as described^42^. LipidSearch (4.2.27) and Compound Discoverer (3.1.0.305) in tandem performed untargeted data analysis. Heatmaps and correlation data were generated by MetaboAnalyst (5.0)^44^. Graphs were produced using GraphPad Prism (9.2.0).

## Results

## General and clinical characteristics of study subjects

COVID-19 (+) patient demographics and comorbidities are described in Table 1. Clinical characteristics, including severity scoring, clinical laboratory values, pre-hospital, and in-hospital drug therapies are described in Table 2.

**Table 1:** Baseline Patient Characteristics.

**Demographics**	
Age (years)	60 (25)
**Sex**	
Male	14 (67%)
Female	7 (33%)
**Race**	
Black or African American	12 (57%)
White	8 (38%)
Other	1 (5%)
**Ethnicity**	
Hispanic	0 (0%)
Non-Hispanic	21 (100%)
**Comorbidities**	
Atrial fibrillation	3 (14%)
Acute kidney injury	1 (5%)
Anemia	3 (14%)
Asthma	2 (10%)
Coronary artery disease	4 (19%)
Congestive heart failure	6 (29%)
Chronic kidney disease	4 (19%)
COPD	5 (24%)
Diabetes	11 (52%)
Kidney transplant	3 (14%)
Hyperlipidemia	5 (24%)
Hypertension	18 (86%)
Immunosuppression	3 (14%)
Obesity	11 (52%)
Sickle cell disease	1 (5%)
Transient ischemic attack	1 (5%)
Ventricular tachycardia	1 (5%)

Data are n (%) or median (IQR).

**Table 2:** Patient clinical characteristics.

**Clinical features**		
BMI	32.6 (13.6)	
History of smoking/tobacco use; n = 20	10 (50%)	
Pregnant	1 (5%)	
SOFA score on admission	1 (2)	
Days in hospital	11 (17)	
Died in hospital	3 (14%)	
**Received blood transfusion during stay**	5 (24%)	
Units received	4 (16)	
**Use of anticoagulants prior to admission**	3 (14%)	
Aspirin	2 (10%)	
Rivaroxaban	1 (5%)	
**Received anticoagulants in hospital**	19 (91%)	
Apixaban	3 (14%)	
Aspirin	2 (10%)	
Enoxaparin sodium	10 (48%)	
Heparin	5 (24%)	
Rivaroxaban	1 (5%)	
Use of immunosuppressants (for transplant)	3 (14%)	

Patient labs on admission	Research subjects	Reference range
**Hematology**		
RBCs (10^6^/µL) n = 21	4.40 (0.68)	M: 4.0–5.7 F: 3.9–5.1
WBCs (10^3^/µL) n = 20	9.7 (6.2)	4.5–11.0
Platelets (10^3^/µL) n = 20	226 (123)	153–367
Hemoglobin (g/dL) n = 20	12.4 (3.8)	M: 12.6–17.4 F: 11.9–15.7
Hematocrit (%) n = 20	37.8 (10.2)	M: 37–50 F: 35–45
MCV (fL) n = 19	89.8 (8.2)	80–96
MCHC (g/dL) n = 19	31.7 (2)	28–33
RDW (%) n = 19	15.0 (3.6)	12–15%
Absolute lymphocyte count (10^3^/µL) n = 18	0.6 (0.6)	1.3–3.5
Absolute neutrophil count (10^6^/µL) n = 19	7.3 (6.1)	1.7–7.3

**Coagulation**		
d-dimer (ng/mL) n = 17	2190 (4125)	≤ 500
PT (s) n = 10	14.0 (3.6)	12.1–15.0
PTT (s) n = 11	29 (11)	25–38
INR n = 10	1.0 (0.4)	2.0–3.0
**Chemistry**		
Sodium (mmol/L) n = 21	137 (4)	136–145
Potassium (mmol/L) n = 21	4.1 (0.3)	3.5–5.1
BUN (mg/dL) n = 21	22 (26.5)	M: 9–20 F: 7–17
Bicarbonate (mmol/L) n = 21	29 (11)	21–30
Bilirubin (mg/dL) n = 19	0.6 (0.3)	0.2–1.3
Creatinine (mg/dL) n = 21	1.04 (0.89)	M: 0.66–1.25 F: 0.52–1.04

Data are n (%) or median (IQR).

### Characterization of extracellular vesicles

**Figure 1:**
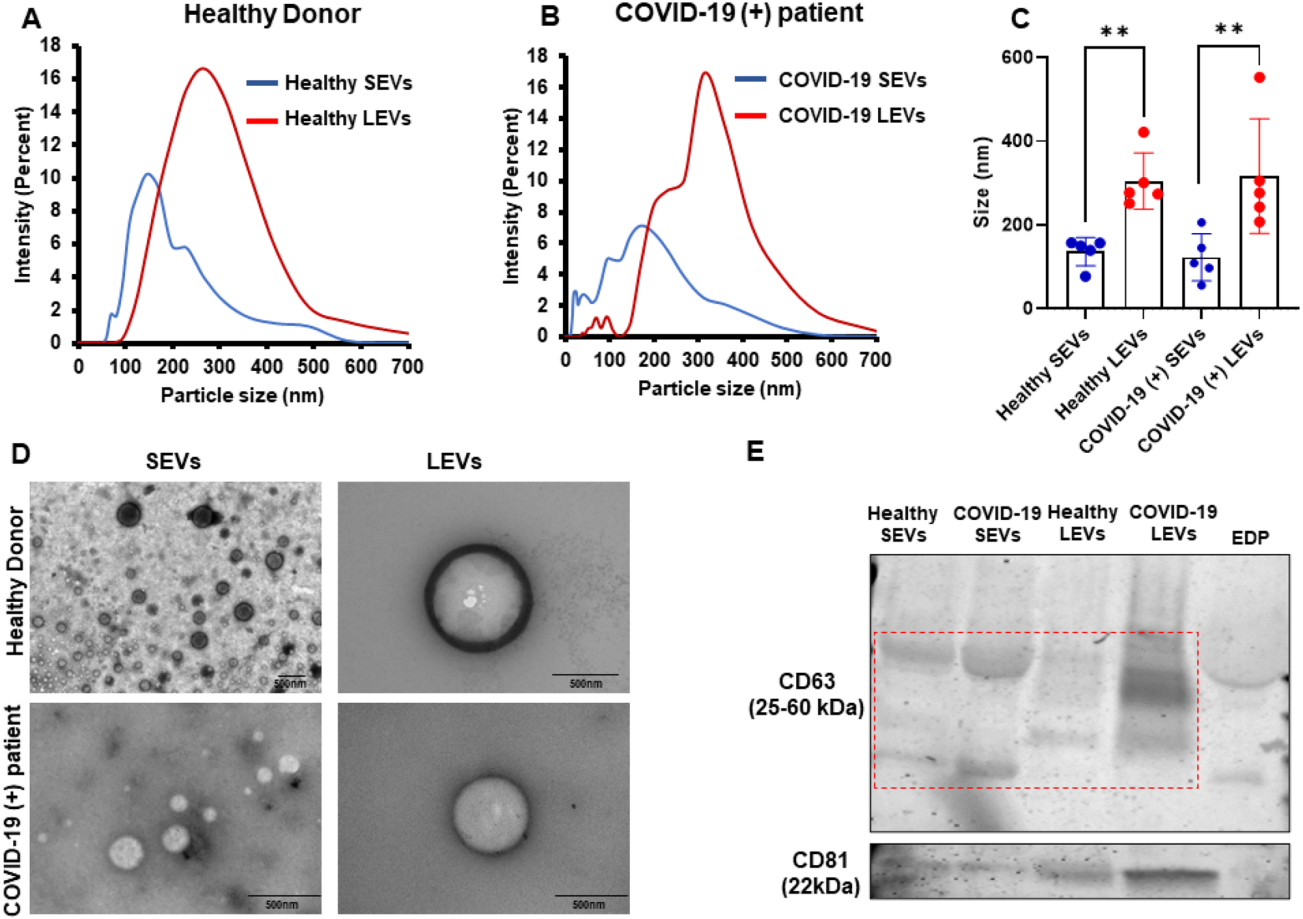
Characterization of EVs: (**A,B**) particle size vs. percent intensity graphs of healthy donor (**A**) and COVID-19 (+) patients (**B**) SEVs/LEVs. (**C**) Mean of n = 5 sample size graph is mentioned. (**D**) TEM images of healthy donor and COVID-19 (+) patients SEVs and LEVs (representative image). An n = 5 each of healthy and COVID-19 SEVs and LEVs was used for particle size and TEM analysis. (**E**) Western blotting analysis of EV specific tetraspanin markers (CD63 and CD81) of healthy donor SEVs/LEVs and COVID-19 (+) SEVs/LEVs and EDP as negative control.

First, we determined the particle sizes of SEVs and LEVs that were isolated from healthy donor and COVID-19 (+) patient plasmas. The average healthy donor SEV size was 136.06 ± 33.66 nm compared to 122.54 ± 56.46 nm for SEV isolated from COVID-19 (+) patients. The average healthy donor LEV size was 304.68 ± 67.53 nm compared to COVID-19 (+) patient average LEV particle sizes of 316.64 ± 136.64 nm. Particle size versus percent intensity graphs of healthy donor and COVID-19 (+) patient SEVs and LEVs are represented in Fig. 1A,B respectively. Figure 1C represents the mean particle size of healthy donor and COVID-19 (+) patient derived SEVs and LEVs. TEM analysis representative images are shown in Fig. 1D reveal visual differences consistent with data obtained from particles sizing. Western blotting analysis (Fig. 1E) displayed EV specific tetraspanin markers (CD63 and CD81) expression of healthy donor SEVs/LEVs and COVID-19 (+) patient SEVs/LEVs and EDP as negative control.

### Flow cytometric detection of extracellular vesicles surface antigens expression

**Figure 2:**
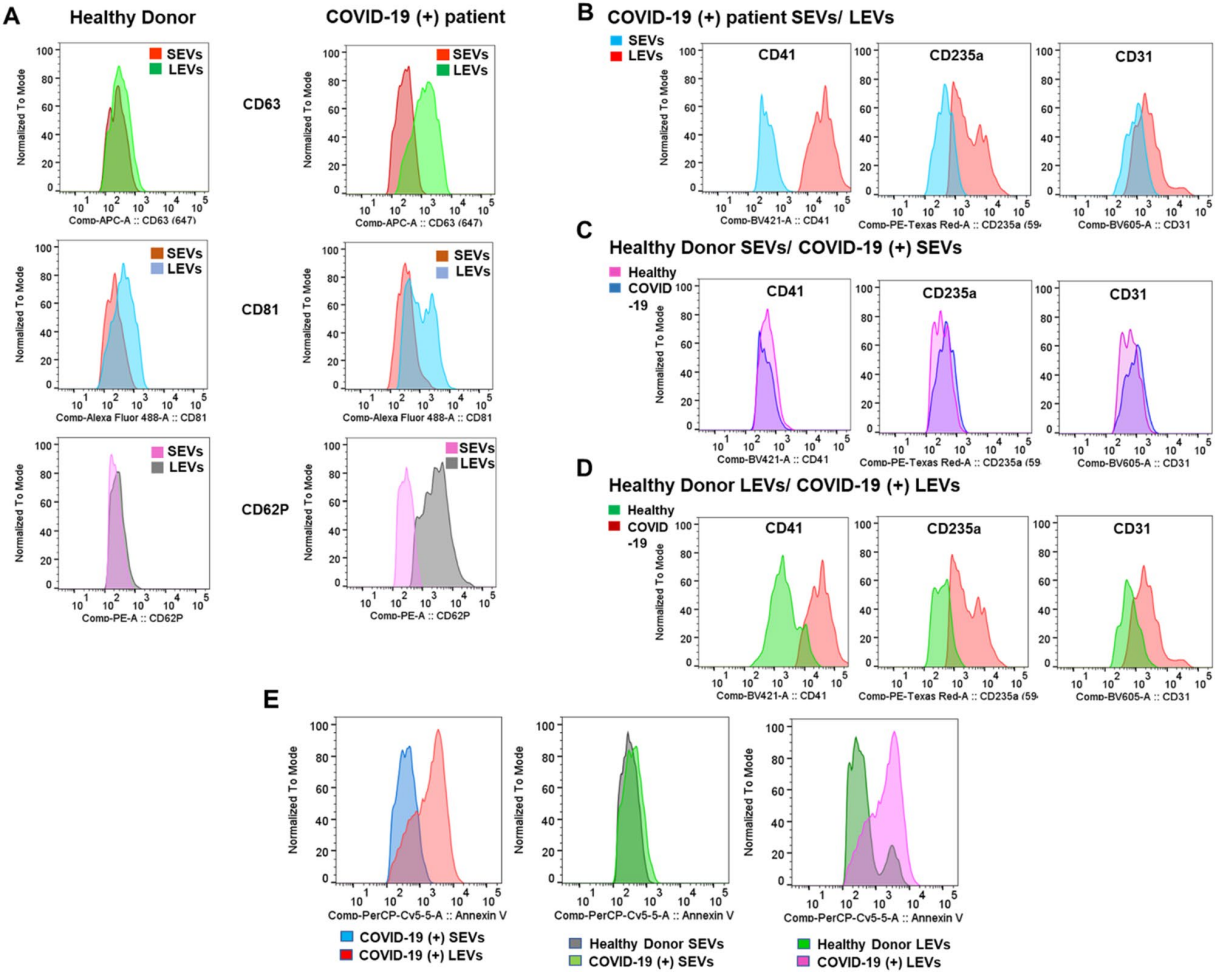
Flow-cytometry analysis of EVs: (**A**) Distribution of CD63, CD81 and CD62P markers in healthy donor (left panel) and COVID-19 (+) patients (right panel) SEVs and LEVs. (**B**) Comparison of CD41, CD235a, and CD31 distribution between COVID-19 (+) patients SEVs and LEVs (top panel) (**C**) healthy donor and COVID-19 (+) patients SEVs (middle panel) (**D**) healthy donor and COVID-19 (+) patients LEVs (bottom panel). (**E**) Annexin V staining between COVID-19 (+) patients SEVs and LEVs (left panel) healthy and COVID-19(+) patients SEVs (middle panel), healthy donor and COVID-19 (+) patients LEVs (right panel). n = 6 each of healthy and COVID-19 SEVs and LEVs used Flow-cytometric characterization.

We next characterized EVs using flow cytometric analysis of SEV and LEV particle-specific markers to confirm measurements made using particle sizing techniques. We used flow cytometry sub-micron particle size reference kit (0.1, 0.2, 0.5 µm) to create the flow cytometry gate for SEV and LEV particles, and the defined gating was applied to all samples as shown in Fig. S2A. Purity of EV depleted plasma (EDP) was determined using the same gate as compared with pooled normal plasma (PNP) (Fig. S2B). The EV specific tetraspanin markers, CD63 and CD81 were expressed on SEVs and LEVs from both healthy donors and COVID-19 (+) patients; however, differential expressions of both CD63 and CD81were observed in COVID-19 (+) patient plasma isolated SEVs and LEVs after normalizing the distributions to their mode. There was a clear positive shift of the LEV histogram when compared to the histogram corresponding to SEVs as shown in Fig. 2A. This data suggests that the mean fluorescent intensity (MFI) of both markers for LEVs were significantly increased (CD63, p = 0.001; CD81, p = 0.023) in COVID-19 (+) patients compared to healthy donor samples (Fig. S5A, B).

The activated platelet marker, p-selectin CD62P was observed to be significantly higher (p = 0.0007) in COVID-19 (+) patient LEVs as compared to SEVs as shown in Fig. 2A. However, there was a minimal shift observed between healthy donor SEVs and LEVs. The histograms for CD41 demonstrate that, COVID-19 (+) patient LEVs demonstrate a positive shift from COVID-19 (+) patient SEVs as well as healthy donor LEVs (Fig. 2B,D). COVID-19 (+) patient LEVs, demonstrates significantly greater CD41 (platelet) expression (MFI) compared to healthy donor SEVs (p = 0.0004) and LEVs (p = 0.0006) as well as SEV (p = 0.00004) from COVID-19 (+) patient plasmas (Fig. S5D). A similar trend was observed in the case of CD235a (erythrocyte) and CD31 (endothelial cell) positive vesicles (Fig. 2B). Conversely, healthy donor and COVID-19 (+) patient SEVs displayed similar origins across platelets, endothelial cells, and erythrocytes (Fig. 2C). Erythrocyte (CD235a) and endothelium (CD31) derived LEVs expression (MFI) were also significantly (CD235a, p = 0.016; CD31, p = 0.020) different between healthy donor and COVID-19 (+) patients (Fig. S5E, F), but the positive shift of CD41 population is more evident (Fig. 2D, S5D). Further, flow cytometric analysis suggests that phosphatidylserine (PS) is enriched in COVID-19 (+) patient LEVs compared to healthy donor LEVs (p = 0.0131). Further, exposure of PS on LEVs is also greater (p = 0.0143) than its exposure on the SEVs isolated from COVID-19 (+) patient plasmas (Fig. 2E, S5G).

### COVID-19 (+) patient LEVs demonstrate increased thrombin generation

**Figure 3:**
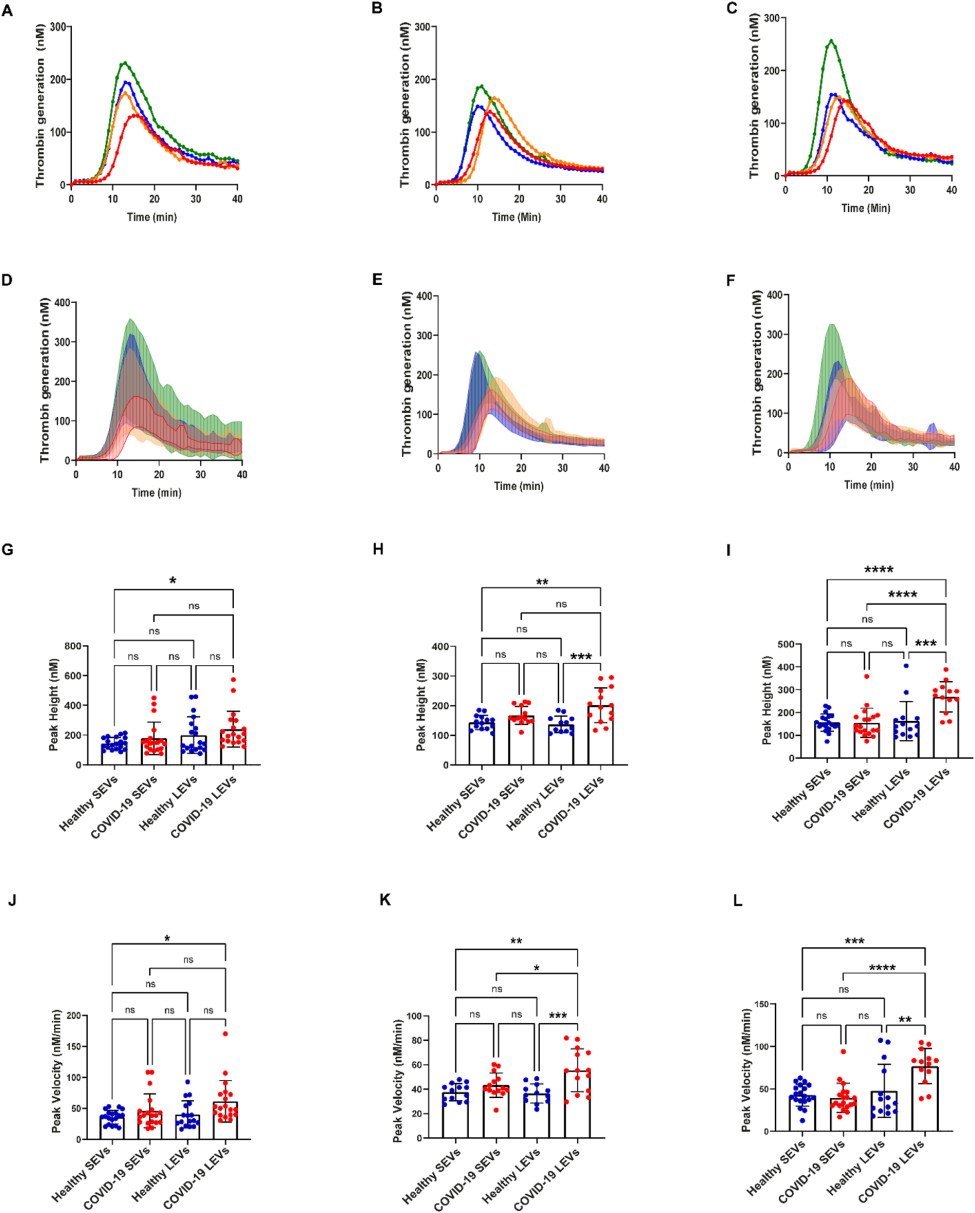
Thrombin generation induced by healthy and COVID-19 EVs: (**A–C**)—mean TG curves for different concentrations of SEVs and LEVs: 50 µg/mL (**A**), 100 µg/mL (**B**), 200 µg/mL (**C**). (**D–F**) Mean thrombin concentrations ± SD over time for (50 µg/mL) (**D**), 100 µg/mL (**E**) and 200 µg/mL (**F**). The lower line for each group represents mean—SD between all the samples in the group, and the upper one represents mean + SD. (**G–I**) Peak height values of healthy donor and COVID-19 (+) patients SEVs and LEVs. (**J–L**) Peak velocity values of healthy donor and COVID-19 (+) patients SEVs and LEVs at 50 µg/mL (**G,J**), 100 µg/mL (**H,K**), 200 µg/mL (**I,L**). 50 µg/mL: healthy donor SEVs (n = 19) LEVs (n = 17), COVID-19 (+) patients SEVs (n = 19) and LEVs (n = 19). 100 µg/mL: healthy donor SEVs (n = 13) LEVs (n = 14), COVID-19 (+) patients SEVs (n = 12) and LEVs (n = 14). 200 µg/mL: healthy donor SEVs (n = 19) LEVs (n = 19), COVID-19 (+) patients SEVs (n = 15) and LEVs (n = 13), EDP (n = 7). Datapoints indicate individual measurements, and p-values are from the one-way ANOVA analysis for comparison between groups. Ns, p > 0.05; *p ≤ 0.05; **p ≤ 0.01; ***p ≤ 0.001; ****p ≤ 0.0001.

To demonstrate the effects of EV isolates on TG, SEVs or LEVs were resuspended in EV depleted plasma at three different concentrations (50, 100, and 200 µg/mL). To date, measurements of TG lag time and time-to-peak are reported to decrease with increasing concentration of EVs isolated from healthy donor plasmas^45^. COVID-19 (+) patient plasma isolated SEVs and LEVs did not show a dose dependent effect on TG. The mean value TG curves are shown in Fig. 3A–C and the mean with standard deviation of all data is plotted separately in Fig. 3D–F for a clearer visual interpretation. Both healthy donor and COVID-19 (+) patient plasma isolated SEVs and LEVs induced TG. Peak velocity and peak height values were calculated as the main parameter of the TG curves as previously reported^40,41^. COVID-19 (+) patient isolated LEVs generate significantly higher thrombin peak heights at 100 µg/mL (p = 0.0005) and 200 µg/mL (p = 0.0004) concentrations, but not at 50 µg/mL (p = 0.6342) when compared to LEVs isolated from healthy donor plasma (Fig. 3G–I). The peak velocity of TG induced by plasma isolated SEVs from healthy donors and COVID-19 (+) patients was not significantly different at the concentrations evaluated (50 µg/mL p = 0.6153; 100 µg/mL p = 0.5562; 200 µg/mL p = 0.9642) as shown in Fig. 3J–L. However, the TG peak velocities observed between healthy donor and COVID-19 (+) patient LEVs demonstrate significant differences at 100 and 200 µg/mL (100 µg/mL p = 0.0006; 200 µg/mL p = 0.0030) concentrations between the two groups as shown in Fig. 3K,L. Further, the thrombin peak velocities generated by COVID-19 (+) isolated LEVs were greater than SEVs (100 µg/mL p = 0.0369; 200 µg/mL p ≤ 0.0001) isolated from the same patients as shown in Fig. 3K,L. Interestingly, this difference was not observed between healthy donor plasma isolated SEVs and LEVs (Fig. 3J–L).

### Extracellular vesicle thrombomodulin, phosphatidylserine, and tissue factor

**Figure 4:**
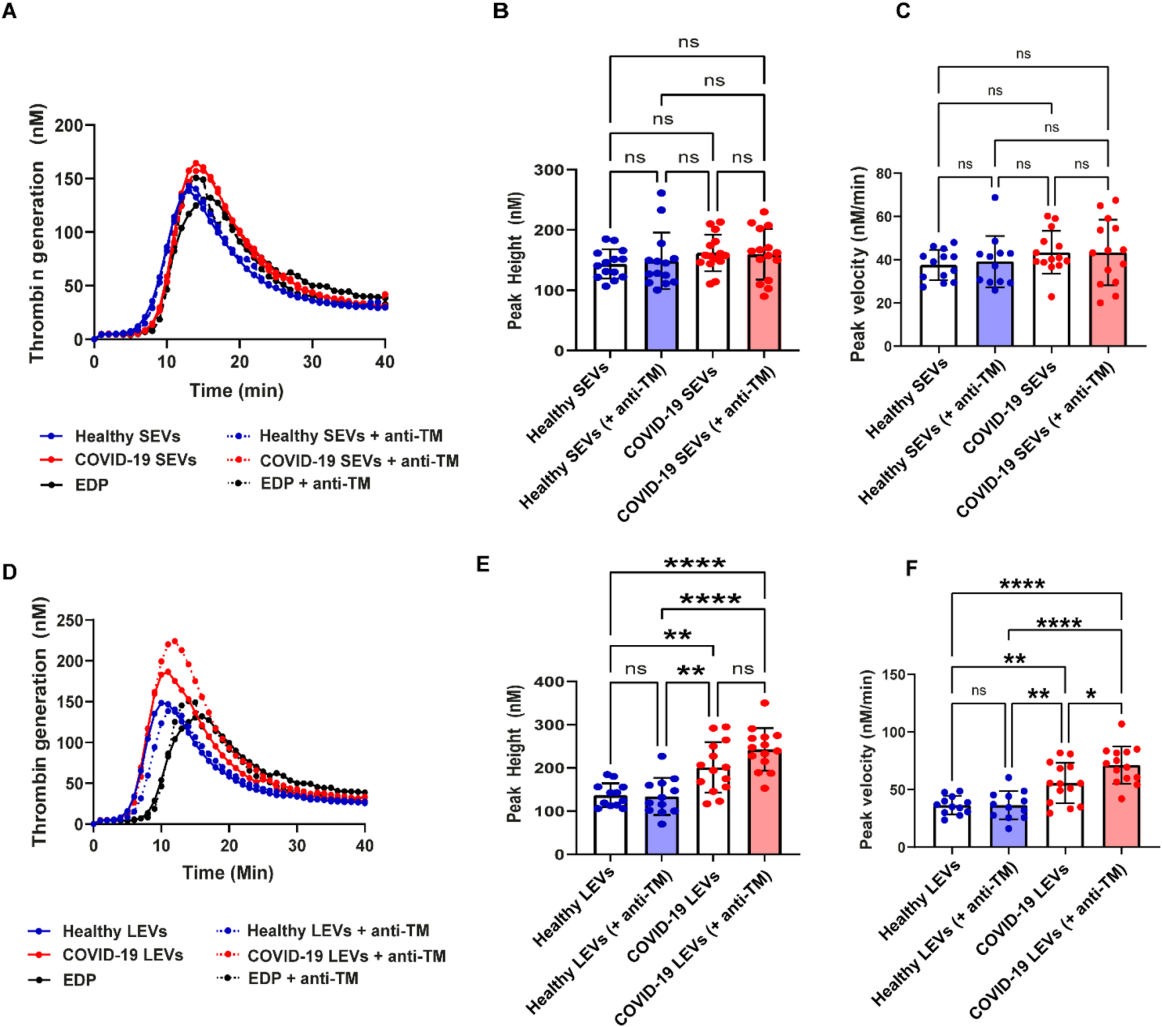
Effect of thrombomodulin on thrombogenicity of SEVs and LEVs. Mean TG curves for healthy donor and COVID-19 (+) SEVs (**A**), and LEVs (**D**) with and without thrombomodulin. Peak height and velocity values of healthy donor and COVID-19 (+) patients SEVs (**B,C**) and LEVs (**E,F**) respectively with and without thrombomodulin antibody. 100 µg/mL of SEVs and LEVs were used for these experiments. healthy SEVs (n = 13) LEVs (n = 14), COVID-19 (+) SEVs (n = 12) and LEVs (n = 14). Datapoints indicate individual measurements, and p-values are from the one-way ANOVA analysis for comparison between groups. ns, p > 0.05; *p ≤ 0.05; **p ≤ 0.01; ***p ≤ 0.001; ****p ≤ 0.0001.

TM is an endothelial cell surface glycoprotein that forms a complex with thrombin and subsequently activates protein C to inactivate FVIIIa and FVa^29^. Therefore, cell surface-bound TM modulates the anticoagulant effect of thrombin. Soluble TM is increased in COVID-19 infection and is a potential marker of endothelial injury^46^. To explore the potential role of TM in COVID-19 (+) patient plasma we isolated SEVs and LEVs and performed blocking experiments to inhibit TM in both SEV and LEV concentrates. Neither healthy donor nor COVID-19 (+) patient plasma isolated SEVs demonstrated changes in TG after anti-TM incubation as shown in Fig. 4A–C. Conversely, both healthy donor and COVID-19 (+) patient plasma isolated LEVs increased TG peak velocities following anti-TM incubation and this effect was significantly (p = 0.0269) greater in COVID-19 (+) patient LEVs compared to all other EV types as shown in Fig. 4D–F.

**Figure 5:**
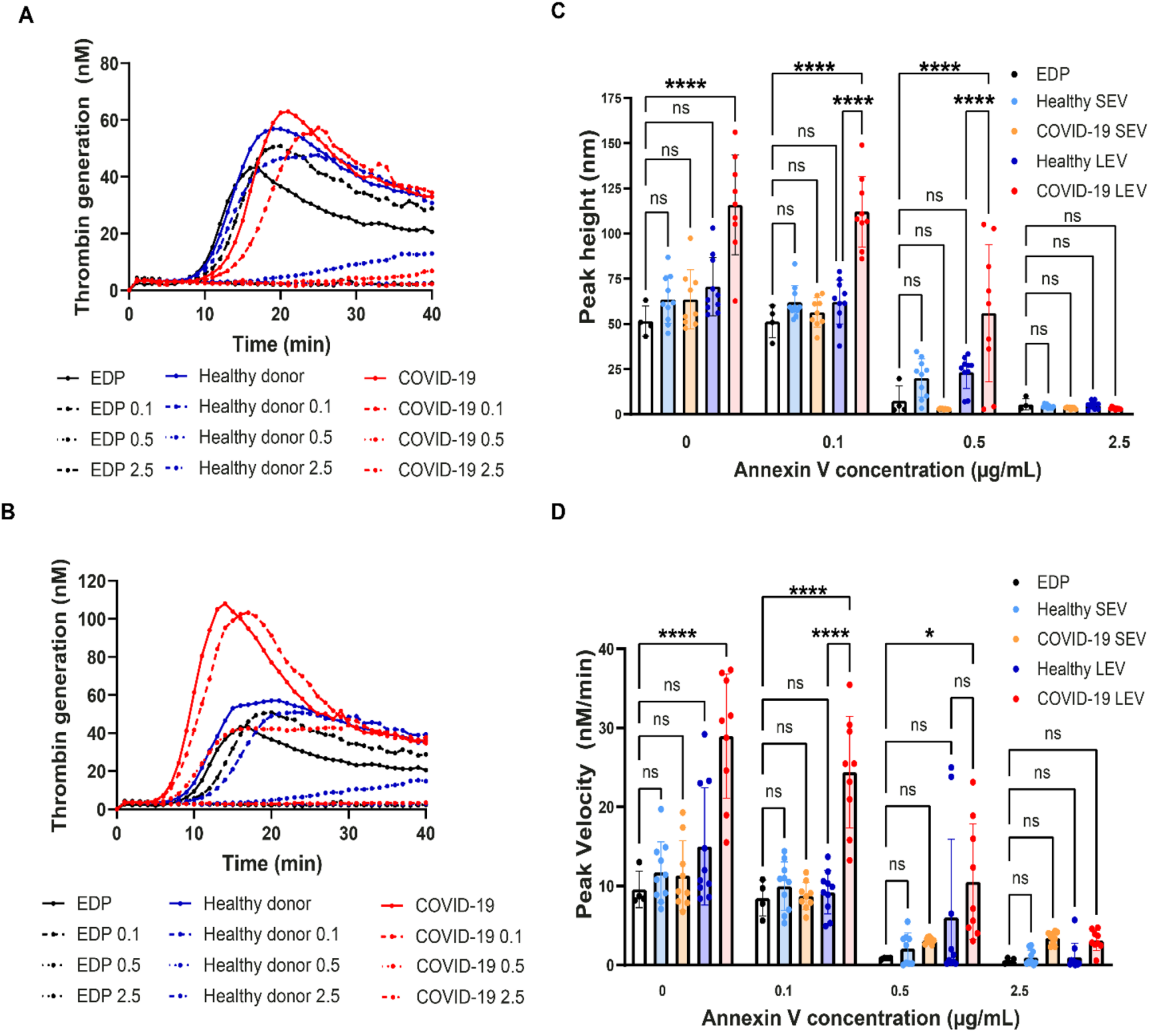
Effect of annexin V on SEV/LEV induced thrombin generation: Mean TG curves for healthy donor and COVID-19 (+) SEVs (**A**), and LEVs (**B**) with different annexin V concentrations. Peak height (**C**), peak velocity (**D**) values of EDP, healthy donor and COVID-19 (+) patients SEVs and LEVs with different annexin V concentration. healthy SEVs (n = 10) LEVs (n = 10), COVID-19 (+) SEVs (n = 9) and LEVs (n = 9).

We next performed EV surface blocking experiments with annexin V at three concentrations (0.1, 0.5, 2.5 µg/mL) to specifically quench the effects of PS mediated TG. Preincubation of healthy donor and COVID-19 (+) patient SEVs and LEVs with annexin V inhibited TG in a dose dependent manner (Fig. 5A,B). Importantly COVID-19 patient LEVs showed increased TG compared to EDP and healthy LEVs at 0.1 and 0.5 µg/mL annexin V concentration (Peak height: p < 0.0001; Peak velocity: 0.1 µg/mL, p < 0.0001). Healthy donor and COVID-19 (+) patient plasma isolated SEVs did not show a significant difference in TG, peak height, and peak velocity at any of the concentrations of annexin V (0.1, 0.5, 2.5 µg/mL) compared to EDP (Fig. 5C,D). In contrast, there were significant differences observed between COVID-19 (+) patient plasma isolated LEVs and EDP for peak height (0.1 µg/mL and 0.5 µg/mL: p < 0.0001) and peak velocity (0.1 µg/mL: p < 0.0001 and 0.5 µg/mL: p = 0.0129) of TG curves at 0.1 and 0.5 µg/mL annexin V concentrations (Fig. 5C,D). At 0.1 µg/mL annexin V both peak height and velocity increased significantly in COVID-19 (+) plasma LEVs compared to healthy LEVs (Fig. 5C,D; p < 0.0001). At 0.1 µg/mL annexin V preincubation, COVID-19 (+) patient LEVs showed TG, but healthy donor LEVs did not (Fig. 5C,D). This suggests that surface accessible PS on COVID-19 (+) patient plasma isolated LEVs is one of the major contributors to TG in the patient samples studied here.

To account for the contribution of TF, we evaluated TF in our healthy donor and COVID-19 (+) patient plasma isolated SEVs and LEVs. Our analysis suggests that TF activity was not different (Healthy SEVs vs. COVID-19 SEVs p = 0.8366; Healthy LEVs vs. COVID-19 LEVs p = 0.3839) between COVID-19 (+) patients and healthy donor plasma isolated SEVs or LEVs (Fig. S6). This observation may be specific to COVID-19 as it contrasts with studies that show TF-activity microparticles across a range of disease states. A TF activity assay for both the SEVs and LEVs did not demonstrate significant difference between COVID-19 (+) patients and healthy donors (Fig. S6).

## Omics characterization of SEV and LEV from COVID-19 (+) patients and healthy donors

**Figure 6:**
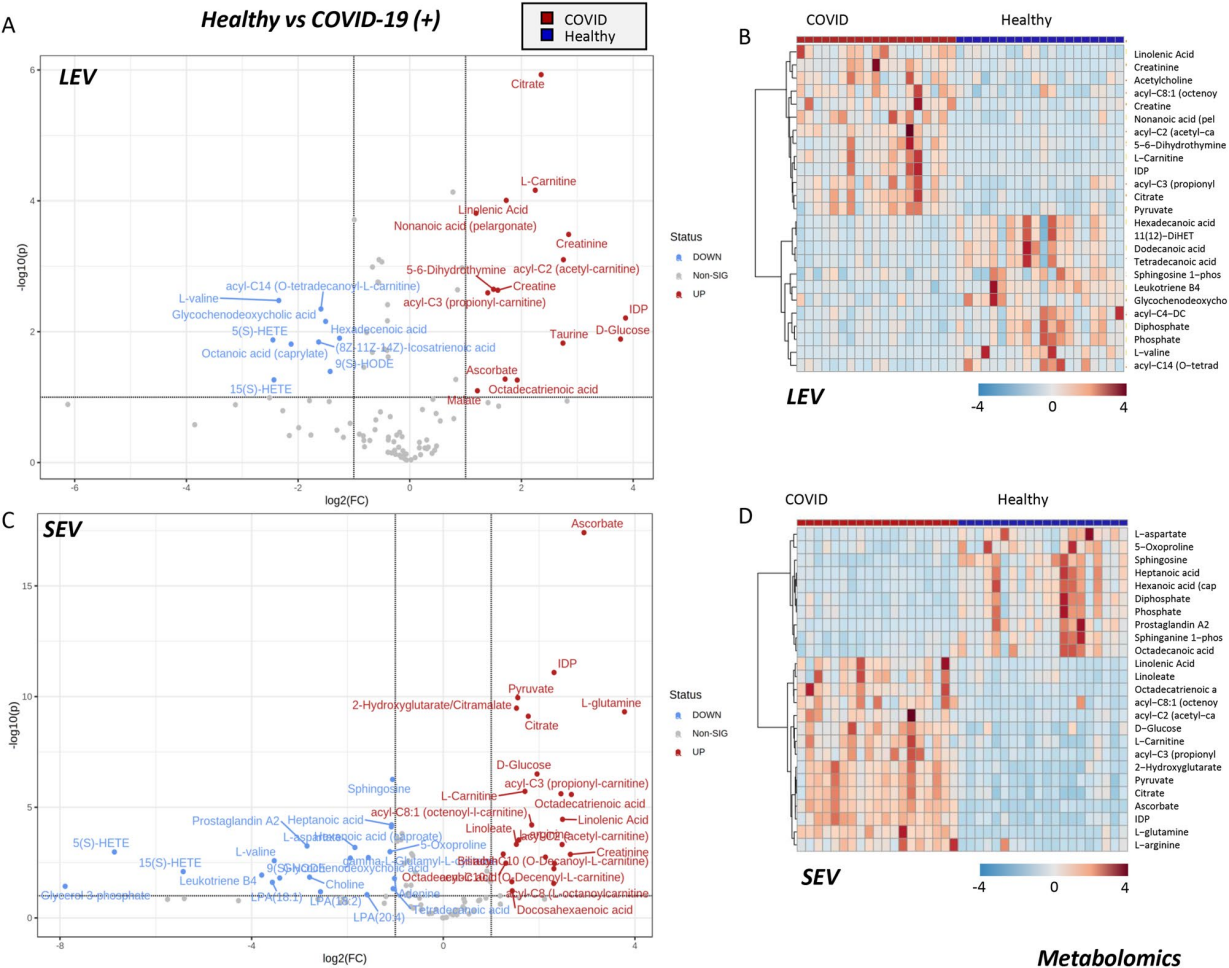
Metabolomics analysis of COVID-19 (+) patient and healthy donor SEVs and LEVs. Volcano plots and heat maps of the most 25 significant metabolites by unpaired two tailed T-test are shown for LEV and SEV in (**A,B**) and (**C,D**), respectively. Heat maps were generated by showing top significant metabolites by T-test (FDR corrected) through the free software MetaboAnalyst 5.0^44^.

**Figure 7:**
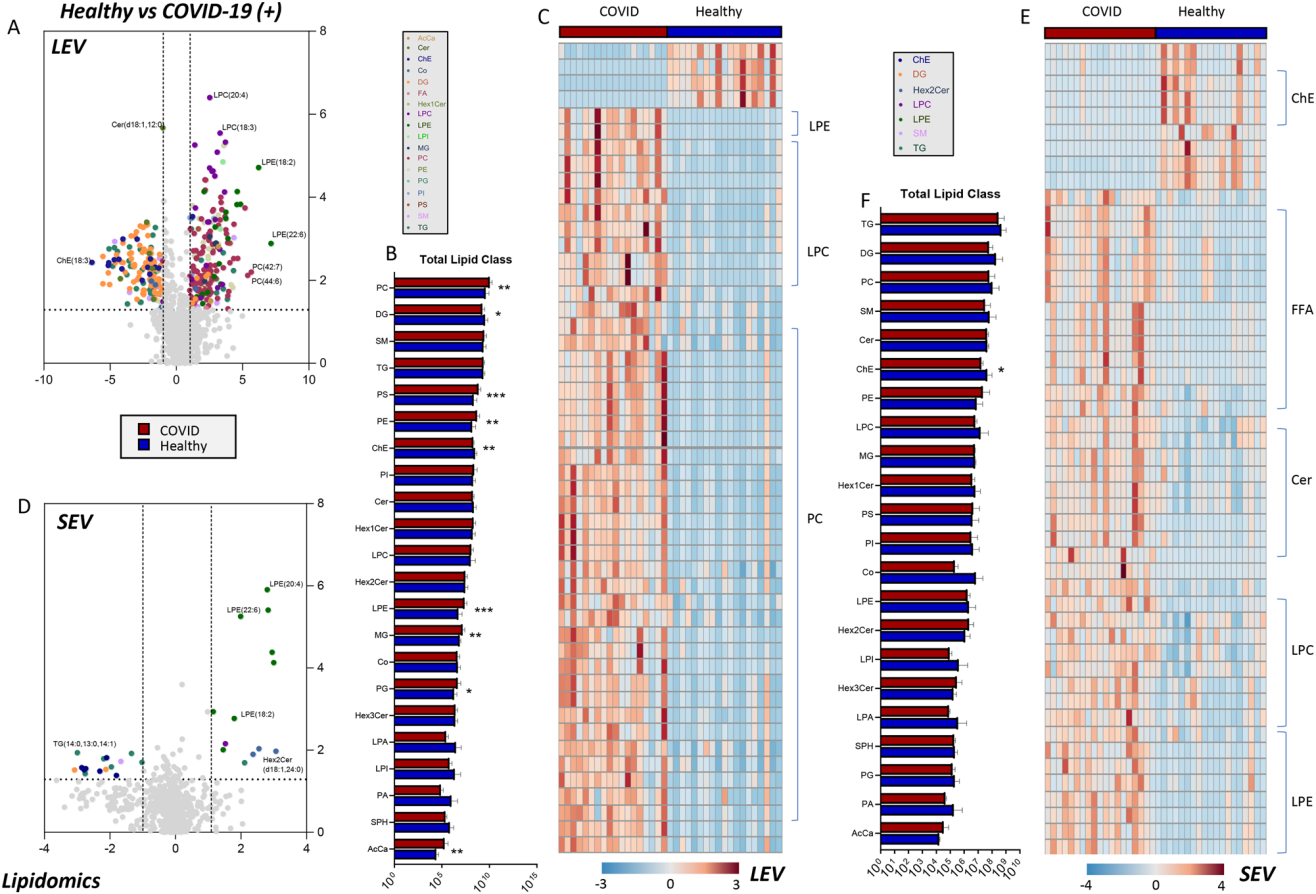
Lipidomic analysis of COVID-19 (+) patient and healthy donor SEVs and LEVs. Volcano plots (color-coded by lipid classes), bar plots (broken down by lipid classes) and heat maps of the top 50 most significant lipids by unpaired two-tailed T-test are shown for LEV and SEV in (A-C and D-F), respectively. Heat maps were generated by showing top significant metabolites by T-test (FDR corrected) through the free software MetaboAnalyst 5.0^44^.

**Figure 8:**
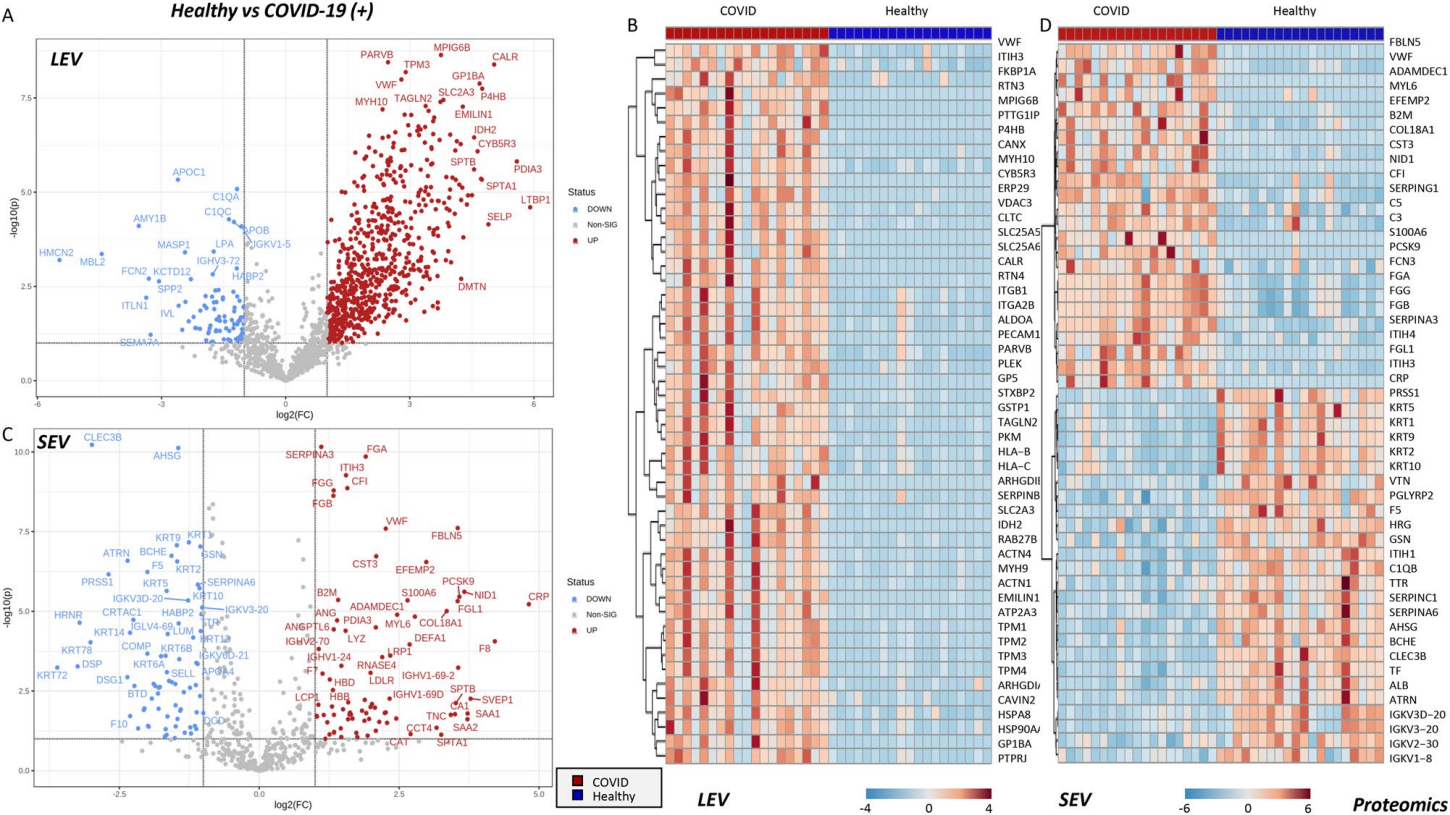
Proteomic analysis of COVID-19 (+) patient and healthy donor SEVs and LEVs. Comparison of proteome of healthy donor and COVID-19 (+) LEVs and SEVs (**A,C** respectively) and unsupervised hierarchical clustering of COVID-19 (+) LEVs and SEVs (**B,D** respectively). Heat maps were generated by showing top significant metabolites by T-test (FDR corrected) through the free software MetaboAnalyst 5.0^44^.

To further understand potential differences in EV populations we performed a multi-omics analysis of SEVs and LEVs from COVID-19 (+) patients and healthy donors, including metabolomics (Fig. 6), lipidomics (Fig. 7), and proteomics (Fig. 8). Results are reported in tabulated form in Supplementary Table 1, for SEVs and LEVs.

Volcano plots and heat maps of the top 25 significant metabolites by unpaired two tailed T-test are shown for LEV and SEV in Fig. 6A–B,C–D, respectively. COVID-19 (+) LEV were enriched in carboxylic acids (citrate, malate), sugar (glucose), antioxidants (taurine, ascorbate) arginine metabolites (creatinine), short-chain acyl-carnitines (C0, C2, C3)—but not medium and long-chain (C14), free saturated fatty acids (C12:0, C14:0, C16:0), sphingolipids (S1P), bile acids (glycochenodeoxycholate), and oxylipins (5-HETE, 15-HETE). Most of these signatures were recapitulated in SEVs as well, especially ascorbate, carboxylic acids (citrate, malate, 2-hydroxyglutarate), and free or acyl-conjugated carnitines.

Given the differences in the lipid species amenable to detection from basic metabolomics analyses, we thus performed an untargeted, more comprehensive analyses of the lipidomes of these samples (Fig. 7). Volcano plots (color-coded by lipid classes), bar plots (broken down by lipid classes), and heat maps of the top 50 most significant lipids by unpaired two-tailed T-test are shown for LEVs and SEVs in Fig. 7A–C,D–F, respectively. Both LEV and SEV from COVID-19 (+) patients were characterized by higher levels of lysophophatidylethanolamines (LPEs), lyosphosphatidylcholines (LPCs), and phosphatidylcholines (PCs). SEV showed also higher levels of unsaturated, long-chain free fatty acids—expanding on metabolomics data—and lower levels of cholesteryl-esters (ChE)—(Fig. 7E).

Of all omics analyses, the most striking differences between LEV and SEV from COVID-19 (+) patients and healthy controls were observed in the proteome (Fig. 8). Supplementary Fig. S7 shows the results of an unsupervised hierarchical clustering of LEV and SEV samples (Fig. 8A,B) and a network view (Fig. S7) of the top 50 most significantly increased proteins in samples from COVID-19 (+) patients, which were enriched in platelet-derived coagulation components. Specifically, VWF, F13A1 (factor XIII A subunit), F13B (factor XIII B subunit), FGA (alpha subunit of fibrinogen), FGB (beta component of fibrinogen), GP1BA (glycoprotein Ib-alpha)—were all procoagulant proteins and/or of platelet origin enriched in both LEVs and SEVs from COVID-19 (+) patients. Other proteins enriched in these groups—like spectrins SPTA1 and SPTB—are suggestive of components of erythrocytic origin. Specific to SEVs, a decrease in multiple keratins and serin-protease inhibitors (SERPINC1, A6, but not G1) was observed in COVID-19 (+) patients (Fig. 8C,D).

### Omics correlates to thrombin generation

**Figure 9:**
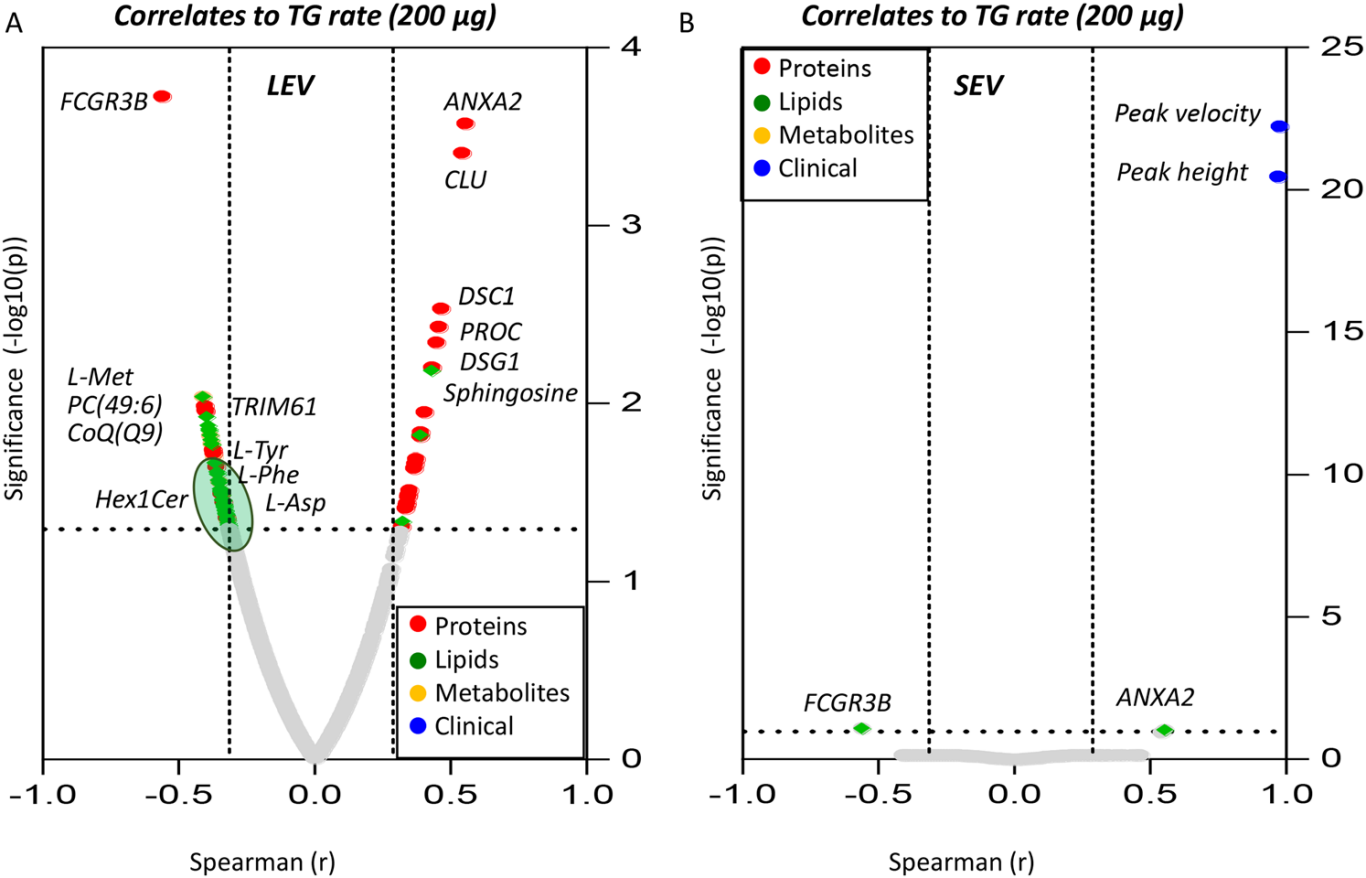
Correlation of TG parameters with omics analyses Spearman correlation analyses was performed to correlate COVID-19 (+) patients TG rate to omics analyses. 200 µg/mL LEV (**A**) and SEV (**B**) data was used.

To tie omics analyses to functional readouts of the procoagulant activity of LEVs and SEVs derived from COVID-19 (+) patients, we performed (Spearman) correlation analyses (Fig. 9A,B, respectively—for the 200 μg dose). Results noted that, while TG parameters (rate, peak height, peak velocity) were strongly positively correlated among each other (r ~ 1 in both LEVs and SEVs), protein components—followed by lipids—showed the strongest correlation to the functional measurements. Of note, several significant (merged) omics correlates to TG rate were identified for LEVs (Fig. 9A), but not SEVs (Fig. 9B)—consistent with a higher procoagulant activity of the former. Several phosphatidylcholines (PCs), triacylglycerols, coenzyme Q9 and ceramides (Hex1Cer) ranked amongst the strongest negatively correlated parameters to TG rate in LEV (Fig. 9A). Similarly, multiple amino acids (aspartate, methionine, phenylalanine, tyrosine) and proteins (TRIM61) negatively correlated with TG rate in LEVs (Fig. 9A), while only proteins (especially ANXA2, CLU, DSC1, PROC, DSG1) and lipids (sphingosine) showed significant positive correlations to TG rate in LEVs (Fig. 9A). ANXA2 levels were positively associated with TG rate in both LEVs and SEVs (Fig. 9A,B).

## Discussion

In this study we compared the thrombogenic potential of SEVs and LEVs isolated from COVID-19 (+) patient and healthy donor plasmas. Our results suggest enrichment for EV specific markers CD63, CD81, and CD62P and procoagulant PS in COVID-19 (+) patient plasma EVs compared to healthy donors. We report for the first time the impact of COVID-19 (+) patient plasma LEVs ability to increase TG and identify metabolic, lipid, and protein correlates consistent with coagulopathy.

EVs play a key role in the pathogenesis of various disease conditions, including COVID-19 infection^18,19^. In this study, SEVs and LEVs were purified from COVID-19 (+) patient and healthy donor plasmas using a polymer-based reagent and sequential centrifugation, respectively. Flow cytometry confirmed the enrichment of EV specific markers CD63, CD81 and CD62P in COVID-19 (+) patient LEVs compared to healthy donors and COVID-19 (+) patient SEVs.

Initially EVs were characterized by their procoagulant activity and termed “platelet dust”^47^. The exposure of anionic phospholipids—especially PS—is an important factor  that contributes to the procoagulant activity of EVs^48–51^. PS exposure on intact platelets and platelet-derived microparticles has been widely studied^48^ and there are several reports on PS exposure on other cell types such as erythrocytes^52,53^ and endothelial cells^54,55^. Different levels of PS^+^ platelet EVs are reported in COVID-19 (+) patients due to the severity of disease^35,56^. To date we know of one study that has implemented flow cytometric evaluation of COVID-19 (+) patient whole blood and reported on elevated levels of CD41 and CD31 specific EVs^30^. Experiments performed to block SEV and LEV surface PS with annexin V confirm an increased PS-mediated TG peak height and velocity caused by LEVs isolated from our COVID-19 (+) patient plasmas. This suggests that PS is an important contributor to LEV-mediated aberrant coagulation in COVID-19. Conversely, TM plays a critical role in anticoagulation and acts to mediate the catalysis of protein C (positively correlated with TG rate in LEVs) activation following thrombin binding. Normally TM is closely associated with the endothelium, but it is reported to be increased in the circulating plasma of COVID-19 (+) patients, presumably due to diffuse endothelial injury consistent with the disease. Here we observed that the TG was greater after TM blocking in COVID-19 (+) patient plasma isolated LEVs compared to LEVs from healthy donors, indicating a loss of TM from the endothelium.

To understand assay measurements of thrombin mediated procoagulant activity of SEVs and LEVs from COVID-19 (+) patients and healthy donors, we also performed a combined metabolomics, lipidomics, and proteomics analysis on the same samples. Results confirmed an enrichment for platelet-derived proteins in the SEVs and LEVs in both groups, with a fraction of EVs likely of erythrocytic origin. Further proteomic analysis revealed VWF, F13A1 (factor XIII A subunit), F13B (factor XIII B subunit), FGA (alpha subunit of fibrinogen), FGB (beta component of fibrinogen), GP1BA (glycoprotein Ib-alpha)—were all procoagulant proteins and/or of platelet origin enriched in both LEVs and SEVs from COVID-19 (+) patients. Of note, positive correlation between the levels of annexin 2 (ANXA2) and TG rate were observed for both LEVs and SEVs, consistent with the role of this peripheral membrane binding protein in lipid segregation and membrane budding^57^. These results are in keeping with and expand on previous omics studies of sera^58^ and red blood cells from COVID-19 (+) patients, the latter showing that oxidative damage to the erythrocyte membrane likely fueled vesiculation of blood cell-derived protein components and complex lipids as a function of disease severity^59^. Omics analyses also confirmed a significant alteration of the lipidome of LEVs from COVID-19 (+) patients, which were found to be enriched with several phospholipid classes (PCs, LPCs, LPEs, with PEs ranking amongst the top correlates to TG parameters with other neutral lipids, e.g., triacylglycerols). Of note, lipidomics analyses of LEVs showed a significant negative correlation between lipid levels (especially triacylglycerols and ceramides) and TG parameters, while higher PS exposure (but not levels) in LEVs was positively associated with it. This observation suggests that a combination of lipidome composition and compartmentalization contributes to the procoagulant effect of LEVs from COVID-19 (+) patients and that PS is more externalized to the LEV surface. Unexpectedly, lower levels of oxylipins were observed in EVs from COVID-19 (+) patients – metabolites that are enriched in mammalian red blood cells and negatively correlate with erythrocyte propensity to undergo splenic sequestration and extravascular hemolysis^60^. Though speculative at this stage, this finding—combined with proteomics results and flow-cytometry data—is suggestive of a lesser erythrocytic component to the EV concentration in the circulating blood of COVID-19 (+) patients compared to healthy donors. Expanding on previous hypotheses that associate the role of blood cell-derived vesicles to the alterations of the circulating lipidome of COVID-19 (+) patients as a function of disease severity, as gleaned by IL-6 levels and pre-existing conditions (e.g., obesity)^11^. Besides EVs serving as a marker for vascular damage they play an important role in the pathogenesis of blood clot formation and are reported to increase TG in several disease states^61^. In this study we observed a significant increase in TG parameters induced by addition of COVID-19 (+) patient plasma isolated LEVs compared to COVID-19 (+) patient SEVs and compared to healthy donor plasma isolated SEVs and LEVs. TG associated with the COVID-19 (+) patients LEV fraction of plasma accumulated EVs in this study supports an observation of increased TG in COVID-19 (+) patient versus acutely ill patient plasmas upon hospital presentation and admission^62^.

This study does have several limitations as follows: all samples from confirmed COVID-19 (+) patients were obtained within 1–3 days of hospital admission which may alter the cargo of both SEVs and LEVs and temporal samples over the course of hospital stay to assess progressive disease were not obtained. Additionally, the data is derived from a small group size (n ≤ 21) for both COVID-19 (+) and healthy donor plasma samples and the COVID-19 (+) patient population demonstrates a wide range of pre-existing health conditions and pre-hospital medications that may have impacted SEV and LEV composition. In this study, we were limited to small volume (4 mL) blood collections and as a result polymer based SEV isolation was our primary option. Further, the addition corn trypsin inhibitor (CTI) to tubes used for blood collections in healthy donor and COVID-19 (+) patients was not logistically possible. Given the limitations, annexin V concentration dependent experiments and lipidomic analyses clearly shows that LEV-PS is an important modifier of TG in COVID-19 (+) patient plasma. Further research should be carried out to determine the role of coagulation factors interaction with LEVs and their role in TG, and this is relevant for other disease states.

In conclusion, this study is the first to report the role of COVID-19 (+) patient plasma isolated SEVs and LEVs on induction of TG. COVID-19 (+) patient plasma derived LEVs were determined to be of platelet, RBC, and endothelial cell origins in plasmas of COVID-19 (+) patients. A notable procoagulant factor on the cumulative LEV population was PS; and capping of this phospholipid effectively reduced LEV induced TG. The presentation of functional TM on the surface of COVID-19 (+) patient plasma isolated EVs suggests an added risk of microvascular coagulation due to TM loss from the endothelium. Other novel findings identify VWF, F13A1, F13B, FGA, FGB, GP1BA consistent with platelet enriched components of LEVs. Further, the omics signature of COVID-19 (+) patient plasma derived LEVs suggests a utility for a simple TG assay to define the early thrombogenic risk in COVID-19 and other acute and chronic diseases with a known component of hemostasis risk.

## Supplementary Information

Supplementary Figures.

Supplementary Table 1.

## Data Availability

The datasets used and analyzed during the current study are available from the corresponding authors on reasonable request.
