# Supplementary material for: Coagulation potential and the integrated omics of extracellular vesicles from COVID-19 positive patient plasma
Source: Sci Rep. 2022 Dec 23;12:22191. doi: 10.1038/s41598-022-26473-8 (PMC9780627; doi:10.1038/s41598-022-26473-8)
Supplement: Supplementary file 1 — Supplementary Figures. [file 41598_2022_26473_MOESM1_ESM.docx]

***
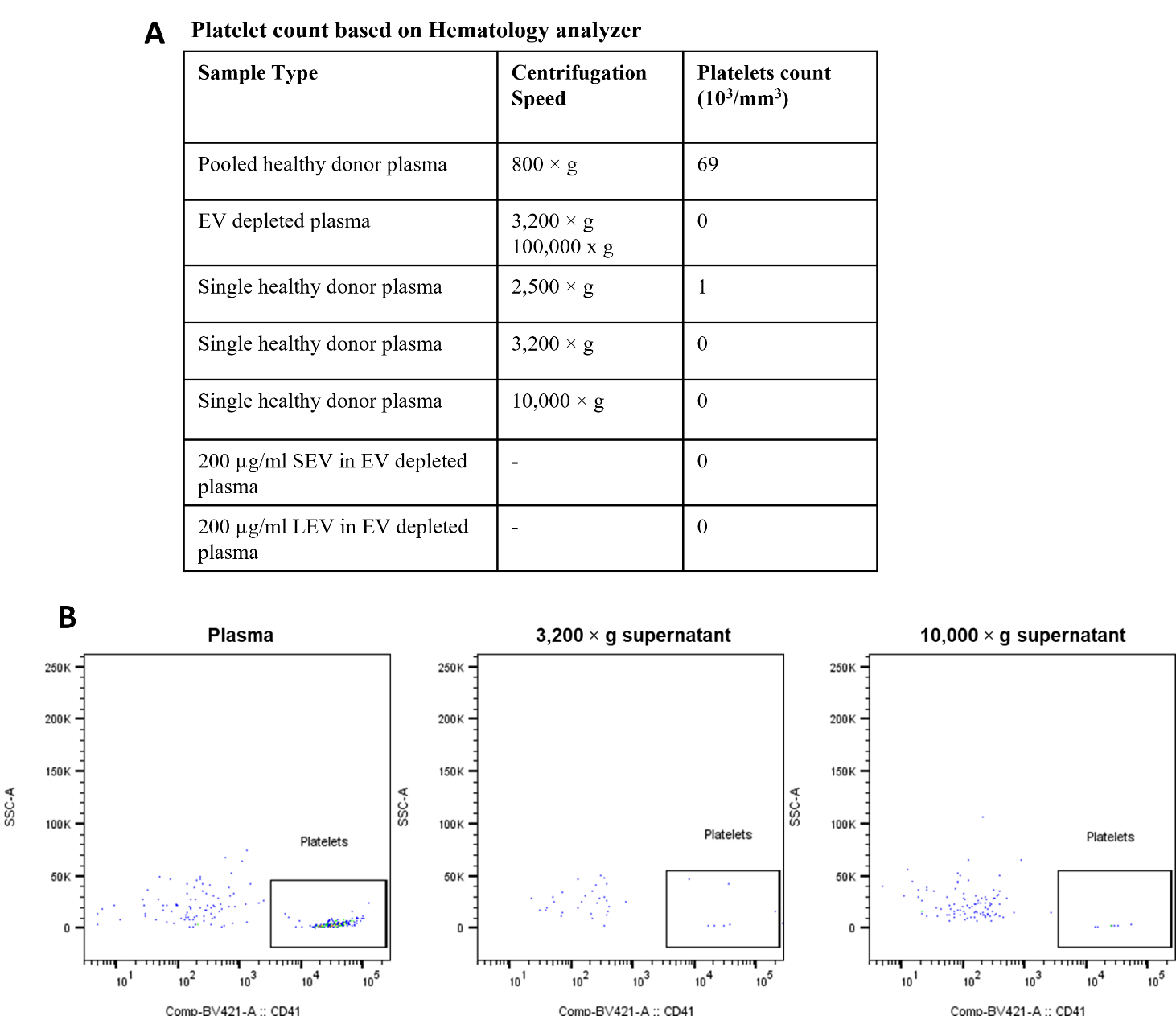
***

***Figure S1 A.*** *Residual platelets count in Platelet poor plasma, EV depleted plasma at different centrifugation steps, SEV and LEV preparations.* ***B.*** *Healthy plasma, 3,200 ×* g *supernatant and 10,000 ×* g *supernatants were analyzed for residual platelets (CD41) by flow cytometry.*


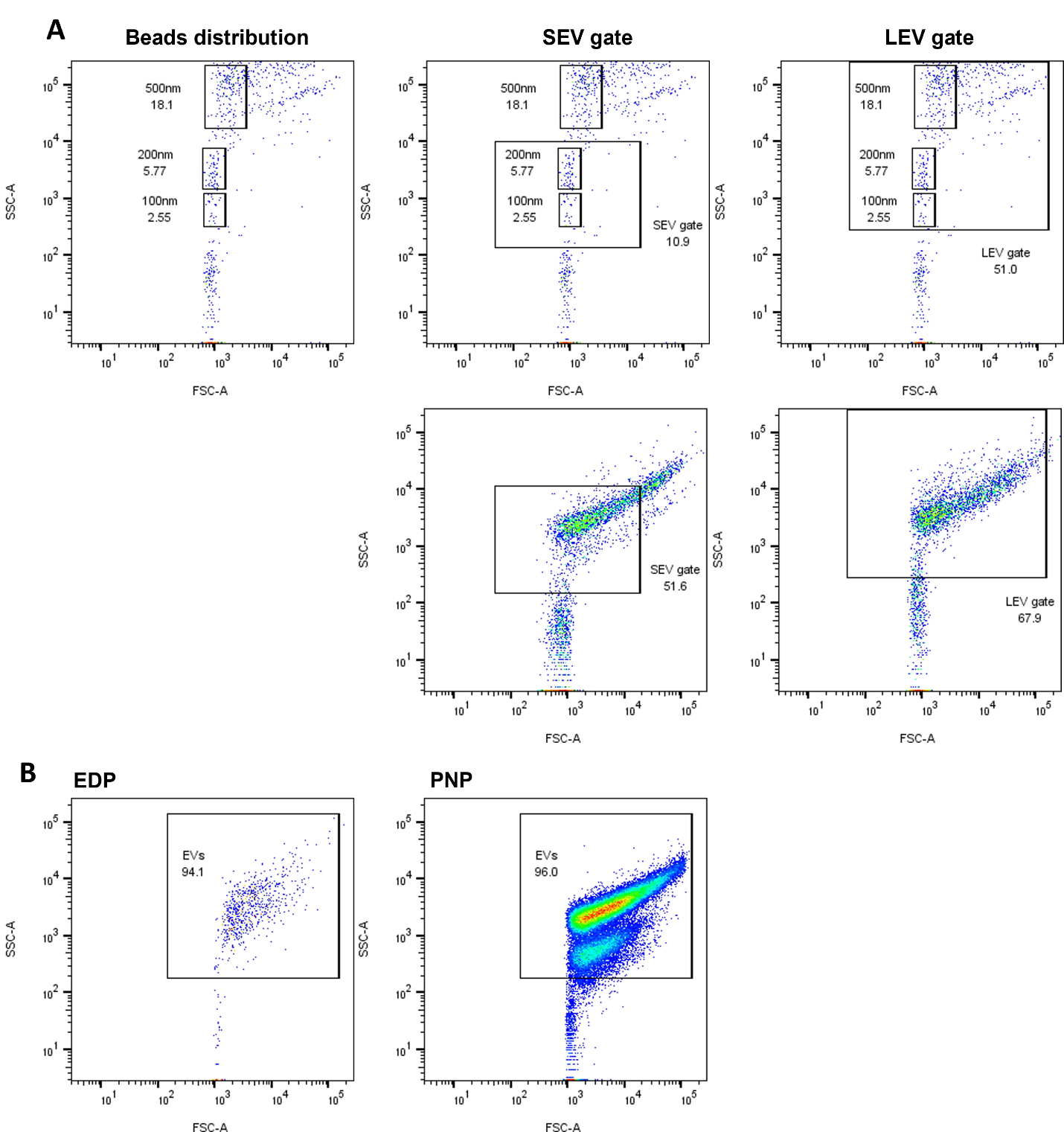


***Figure S2 Flow-cytometry analysis of EVs: A.*** *Beads distribution of flow cytometry sub-micron particle size reference kit (0.1, 0.2, 0.5 µm) and gating strategy of SEVs and LEVs.* ***B.*** *Gating of EV depleted plasma (EDP) (left) and pooled normal plasma (PNP) (right panel).*

*
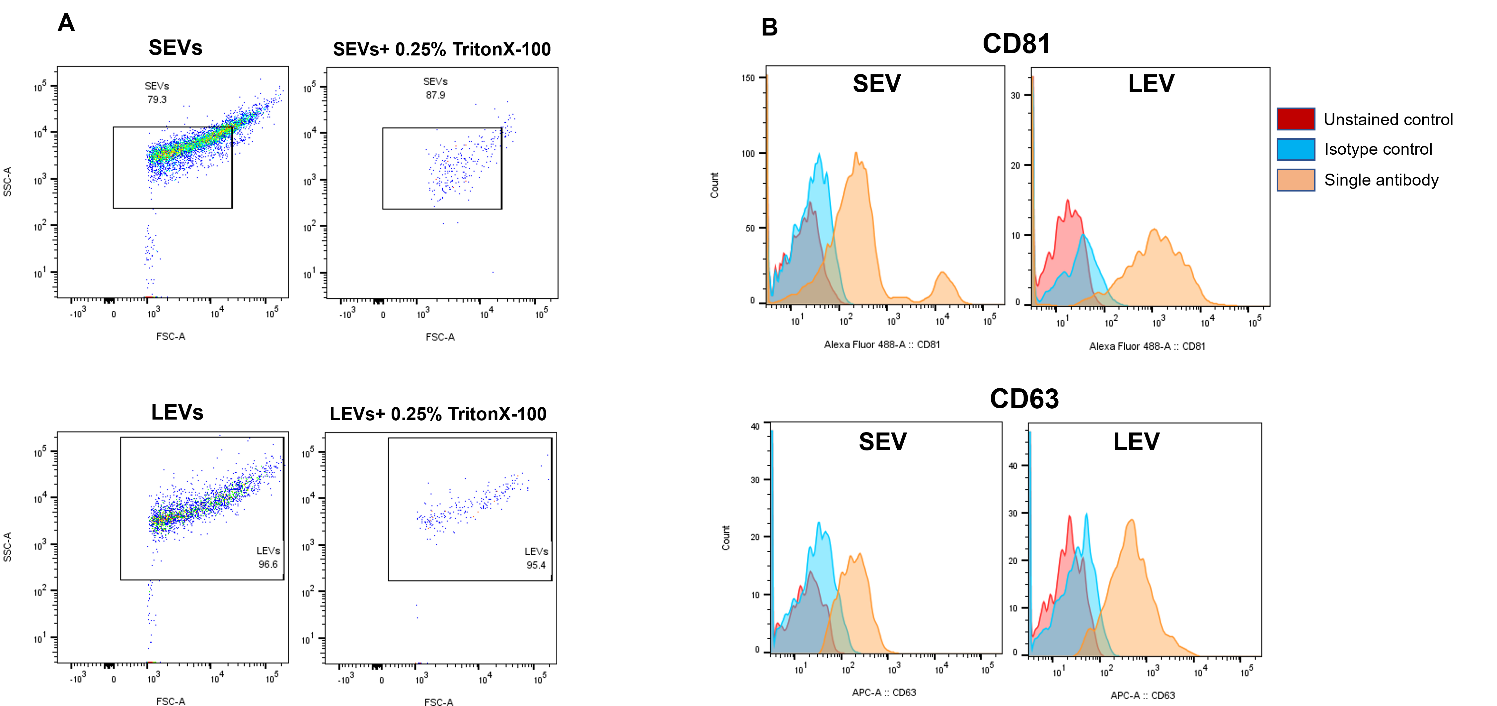
*

***Figure S3 Control experiment for Flowcytometric analysis of EVs. A)*** *Detergent lysis control experiment was performed by treated the SEVs and LEVs with 0.25% TritonX-100 during the staining process. Quality of the particles has been verified by detergent treatment (0.25% TritonX-100) as TritonX could abolish most of the SEVs and LEVs population in the gate. This confirms the presence of intact SEV and LEV population.* ***B)*** *Isotype control experiment along with unstained and single staining of EV specific tetraspanin markers (CD81 and CD63) for healthy SEVs and LEVs.*


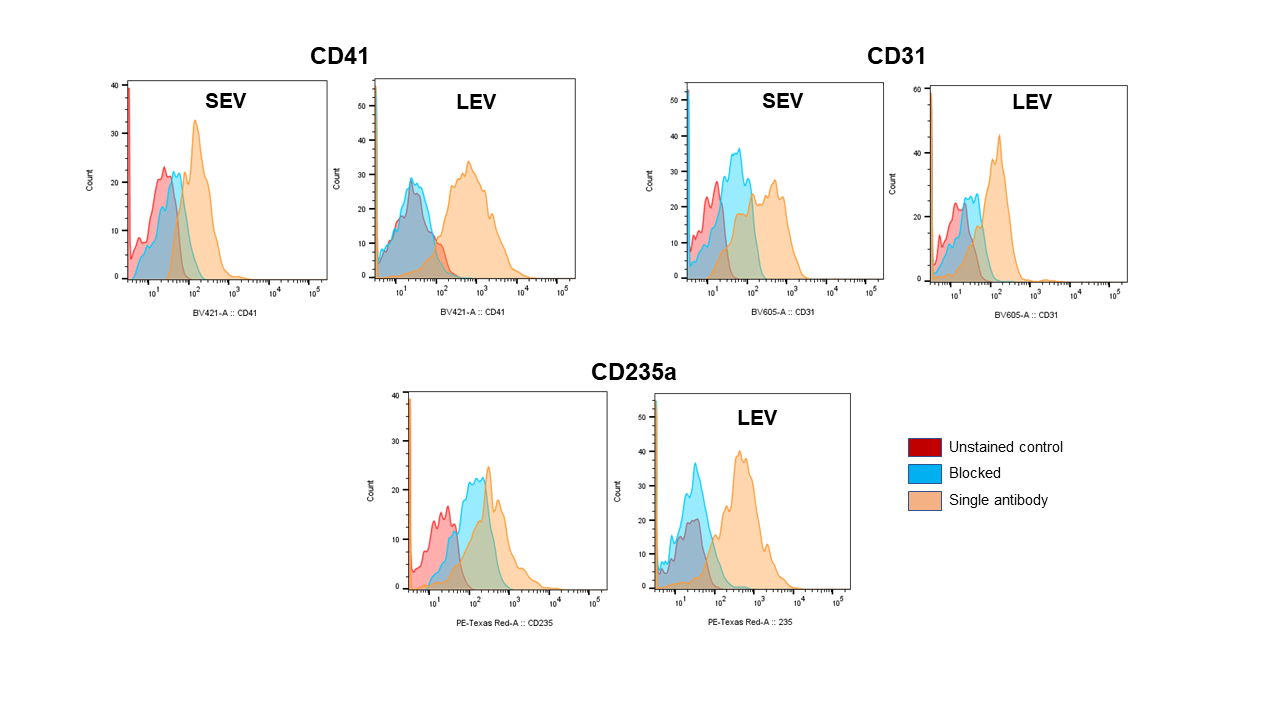
***Figure S4 Control experiment for Flowcytometric analysis of EVs. A)*** *The Blocking experiment has been performed to verify the true expressions of the cell specific antibodies. Both healthy SEVs and LEVs showed cell specific marker (CD41, CD31, CD235) expression.*

***
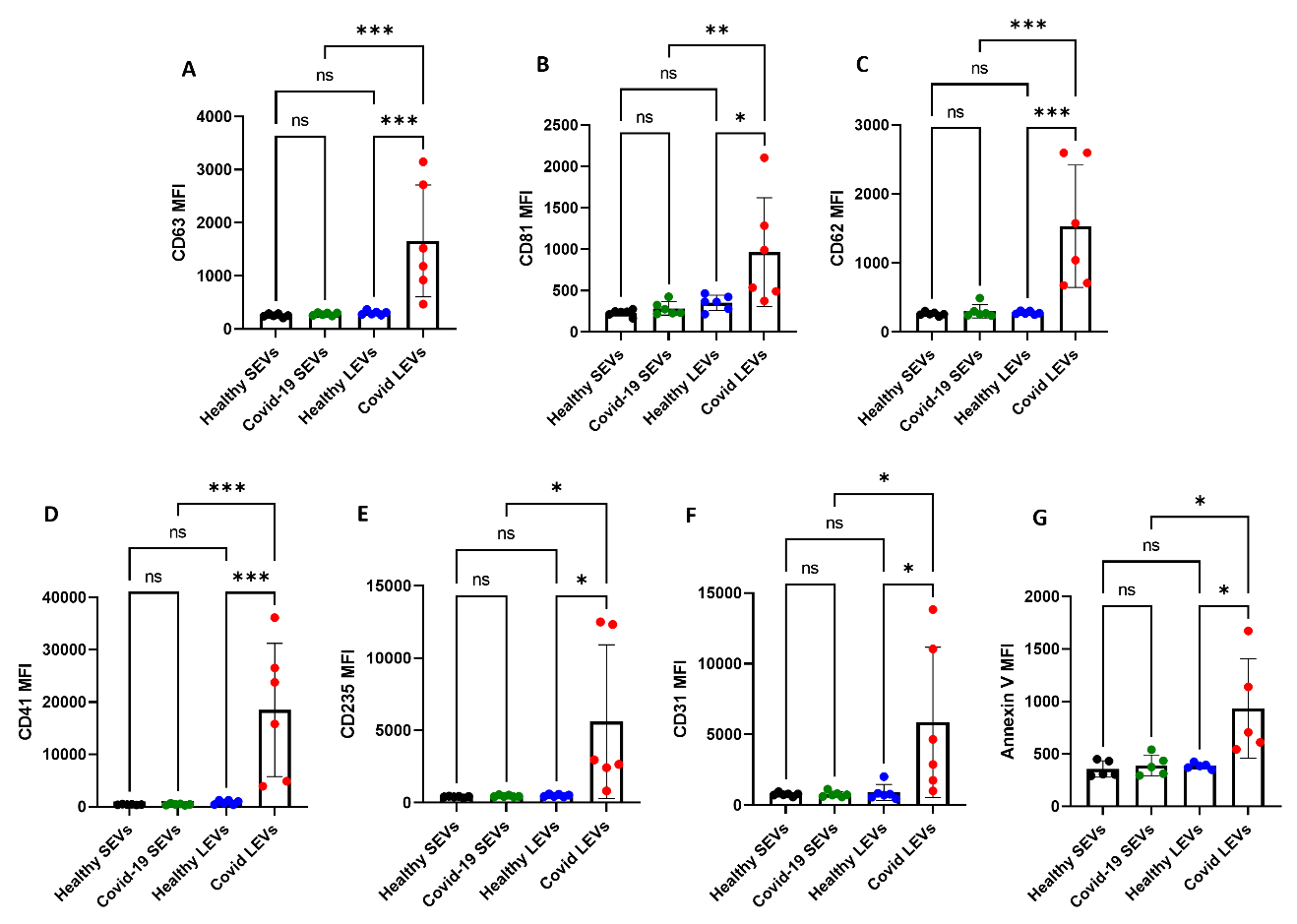
***

***Figure S5 Mean fluorescence intensity of EVs.*** *Comparison of mean fluorescence intensity of different markers* ***(A)*** *CD63,* ***(B)*** *CD81,* ***(C)*** *CD62,* ***(D)*** *CD41,* ***(E)*** *CD235a,* ***(F)*** *CD31 and* ***(G)*** *Annexin V between healthy donor and COVID-19 (+) SEVs and LEVs. Mean of n=6 for all the samples.*

*
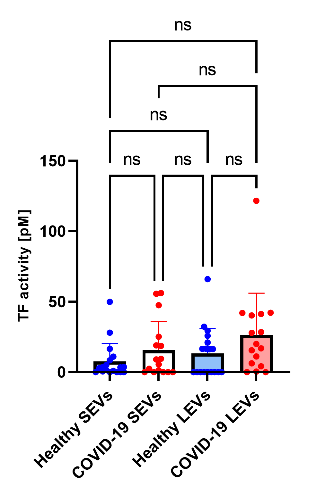
*

***Figure S6 Tissue factor activity of SEVs and LEVs:*** *Tissue factor activity was measured for healthy donor and COVID-19 (+) SEVs and LEVs (n=18). Datapoints indicate individual measurements, and p-values are from the one-way ANOVA analysis for comparison between groups. ns, P > 0.05; *P ≤ 0.05; **P ≤ 0.01; ***P ≤ 0.001; ****P ≤ 0.0001.*

*
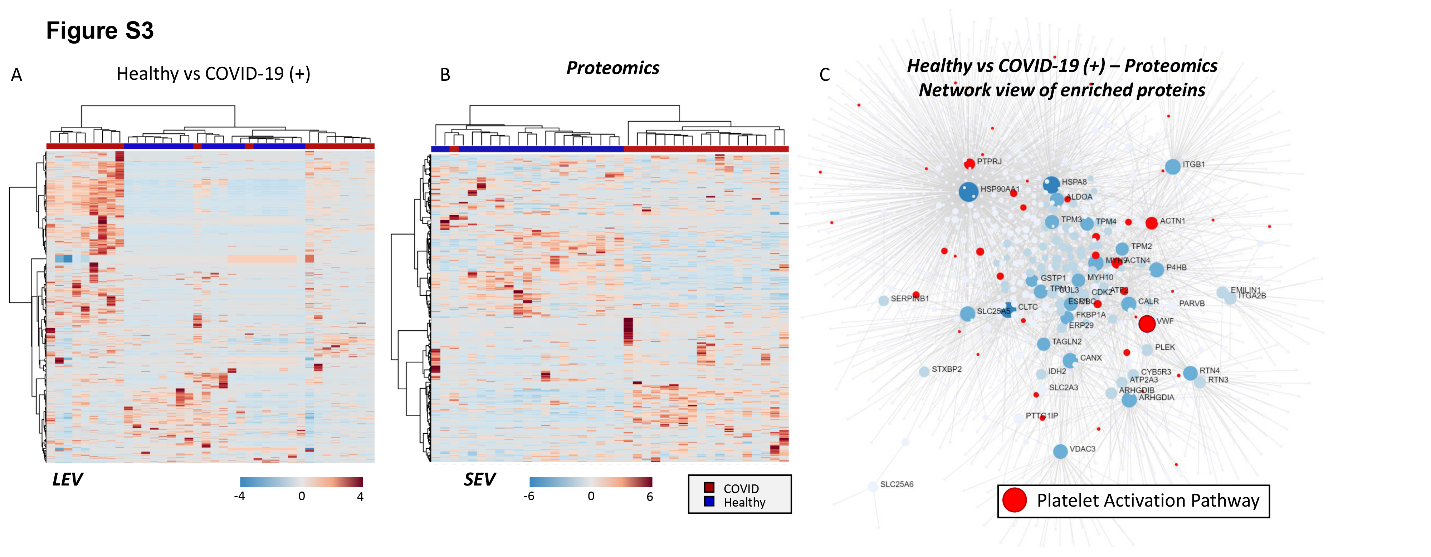
*

***Figure S7*** *An unsupervised hierarchical clustering of LEV and SEV samples* ***(A and B)*** *and a network view* ***(C)*** *of the top 50 most significantly increased proteins in samples from COVID-19 (+) patients* *and healthy donors.*

***
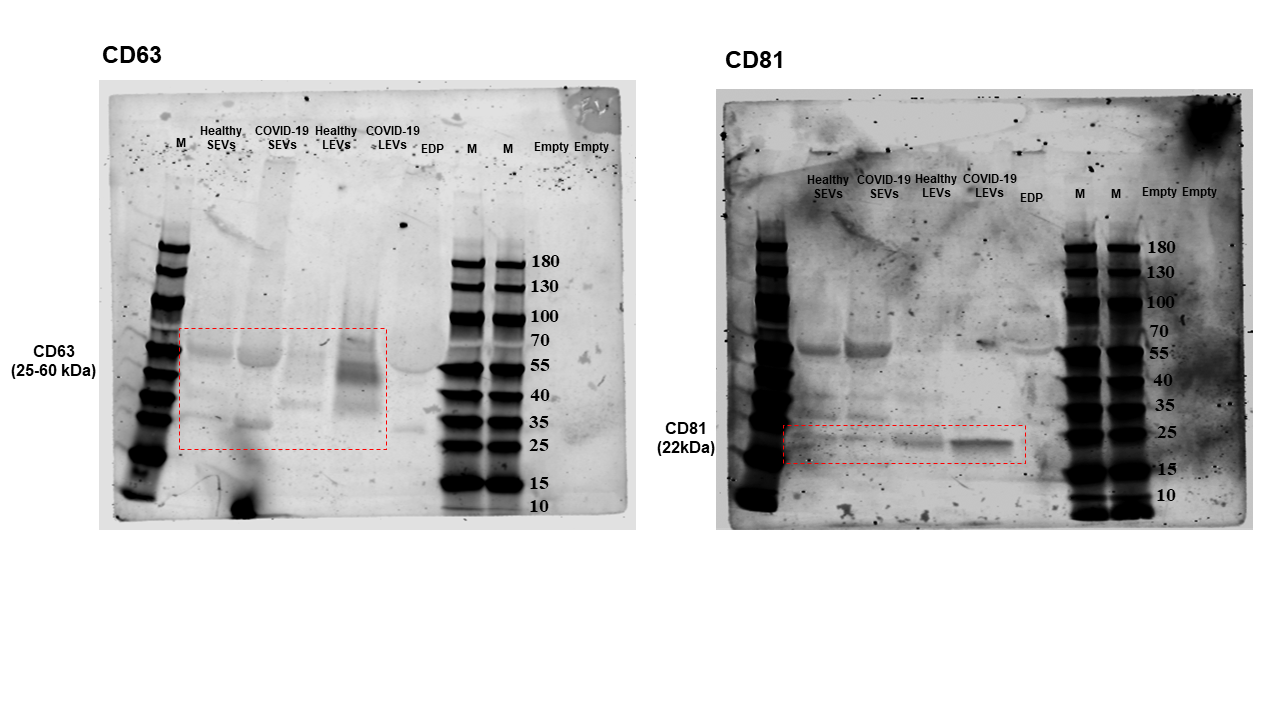
Figure 1 Characterization of EVs****:*

***E)*** *Western blotting analysis of EV specific tetraspanin markers (CD63 and CD81) of healthy donor SEVs/LEVs and COVID-19 (+) SEVs/LEVs and EDP as negative control. M- Protein ladder*
